# Metformin inhibits mitochondrial complex I in intestinal epithelium to promote glycaemic control

**DOI:** 10.1038/s42255-026-01530-y

**Published:** 2026-05-08

**Authors:** Zachary L. Sebo, Ram P. Chakrabarty, Rogan A. Grant, Karis B. D’Alessandro, Alec R. Koss, Jenna L. E. Blum, Shawn M. Davidson, Colleen R. Reczek, Navdeep S. Chandel

**Affiliations:** 1 Department of Medicine, Division of Pulmonary and Critical Care Medicine, Northwestern University Feinberg School of Medicine, Chicago, IL USA; 2 Department of Biochemistry and Molecular Genetics, Northwestern University Feinberg School of Medicine, Chicago, IL USA; 3 Chan Zuckerberg Biohub, Chicago, IL USA

**Keywords:** Energy metabolism, Type 2 diabetes, Target identification, Metabolism, Pharmacodynamics

## Abstract

Metformin is a versatile biguanide drug primarily prescribed for type II diabetes. Despite its extensive use, the mechanisms underlying its clinical effects, including attenuated postprandial glucose excursions and elevated intestinal glucose uptake, remain unclear. Here we map these and other effects of metformin to intestine-specific mitochondrial complex I inhibition. Using human metabolomic data and an orthogonal genetics approach in male mice, we demonstrate that metformin suppresses citrulline synthesis, a metabolite generated exclusively by small intestine mitochondria, and increases GDF15 by inhibiting the mitochondrial respiratory chain at complex I. This inhibition co-opts the intestines to function as a glucose sink, driving the uptake of excess glucose and its conversion to lactate and lactoyl-phenylalanine. We also find that glucose lowering by metformin is due to repeated bolus exposure rather than a cumulative chronic response. Notably, the efficacy of phenformin, another biguanide, and berberine, a structurally unrelated nutraceutical, similarly depends on intestine-specific mitochondrial complex I inhibition, underscoring a shared therapeutic mechanism.

## Abstract (web summary)

Metformin is shown to exert its glucose-lowering and additional clinical effects through inhibition of mitochondrial complex I in the intestinal epithelium in mice.

## Main

Metformin is the most widely prescribed medication for type II diabetes and the only US Food and Drug Administration (FDA)-approved drug of the biguanide class^1^. Before the 1990s, metformin was thought to promote glycaemic control primarily by enhancing glucose utilization in peripheral tissues, including the intestines, with inhibition of endogenous glucose production considered secondary^2^. However, due to technological advances in isotope tracing and nuclear magnetic resonance, subsequent studies demonstrated a suppressive effect of metformin on endogenous glucose production^3,4^, which is now generally attributed to the direct inhibition of hepatic gluconeogenesis^1,5^. Metformin was initially proposed to elicit its anti-gluconeogenic effect by inhibiting mitochondrial complex I of the electron transport chain in hepatocytes^6,7^. However, this explanation has been refuted because inhibiting complex I requires millimolar concentrations of metformin, which are only attained in the intestines of patients on standard dosing regimens^8–10^. Consequently, alternative molecular targets within the liver have been put forward to explain how metformin suppresses gluconeogenesis^11–13^. In contrast, other studies have challenged the centrality of the liver in metformin’s mechanism of action, demonstrating that metformin does not reduce endogenous glucose production in patients with prediabetes or those with recent-onset or well-controlled type II diabetes^14–17^. Instead, the primary antidiabetic effect of metformin in these patient groups is enhanced glucose clearance^14,15^, concomitant with elevated aerobic glycolysis^14^. Indeed, metformin has consistently been shown to enhance glucose utilization in humans^18–23^.

Mostly due to advances in clinical imaging techniques, it is now clear that the intestines are the primary site of increased glycolytic activity, where metformin promotes both glucose uptake (as measured by ^18^F-fluorodeoxyglucose (FDG) accumulation) and lactate production^8,21,24–27^. ^18^F-fluorodeoxyglucose positron emission tomography (FDG-PET), a widely used imaging method for cancer detection, relies on the elevated uptake of FDG in tumours relative to normal tissues. Because metformin increases FDG accumulation in the small and large intestines, it can obscure tumours during imaging. Consequently, by the early 2010s, discontinuing metformin before FDG-PET scans became standard of care to avoid compromising cancer detection^28,29^.

Despite this clinical progress, both the relative contribution of the intestinal effects of metformin on glycaemic control and the underlying mechanism remain unclear. To address this gap in understanding, a bona fide molecular target of metformin in the intestines must, at minimum, explain the drug’s ability to enhance intestinal glucose utilization and increase blood glucose clearance. Ideally, the inhibition of this molecular target would also account for multiple other clinical effects of metformin.

In this study, we leverage publicly available metabolomic data from humans and genetic tools in mice to pinpoint mitochondrial complex I as an essential therapeutic target of metformin in the intestinal epithelium. In addition to enhanced intestinal glucose utilization and blood glucose clearance, this mechanism accounts for metformin-induced citrulline depletion, improved postprandial glycaemia, and elevated lactoyl-phenylalanine (Lac-Phe) and growth differentiation factor 15 (GDF15) levels—all of which are definitive clinical outcomes caused by metformin treatment. We further determined that phenformin, another biguanide, and berberine, a natural compound used as an over-the-counter treatment for type II diabetes, lower blood glucose through the same mechanism. Thus, we identify mitochondrial complex I in intestinal epithelium as a shared and essential therapeutic target for metformin, phenformin and berberine.

## Results

### Metformin suppresses intestinal citrulline synthesis by inhibiting mitochondrial complex I

**Fig. 1:**
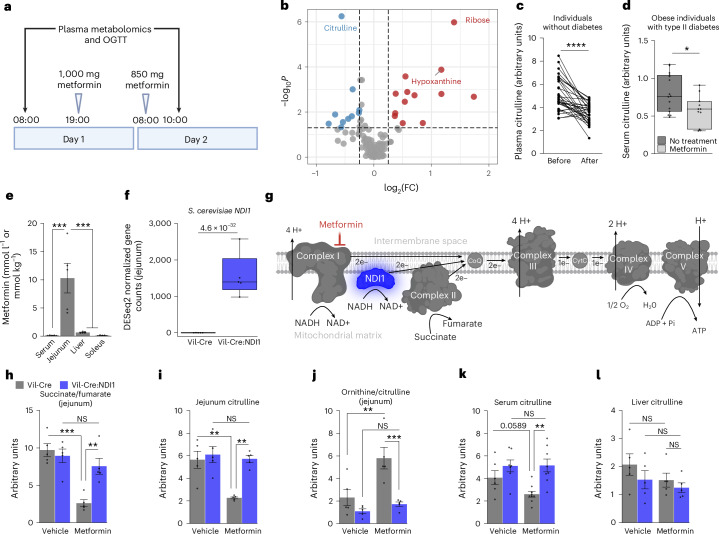
Metformin inhibits mitochondrial complex I in intestinal epithelium to suppress citrulline synthesis. **a**, Schematic illustrating sample collection and metformin dosing in the Rotroff et al.^35^ cohort; related to **b** and **c**. **b**, Volcano plot of 155 known metabolites in blood plasma before and after metformin from the Rotroff et al. cohort of human study participants; metabolites downregulated by metformin are in blue; metabolites upregulated by metformin are in red; the top three metabolites are labelled. *n* = 33. **c**, Plasma citrulline levels in the Rotroff et al. cohort before and after metformin. *n* = 33. **d**, Serum citrulline levels in the Aleidi et al.^36^ cohort of patients with obesity and type II diabetes. *n* = 15 no treatment, *n* = 11 metformin. **e**, Metformin concentrations in serum and different organs of overnight-fasted C57BL/6J mice 1 h after a single oral dose of metformin (200 mg per kg body weight). *n* = 5. **f**, *Ndi1* normalized gene expression in the jejunum of Vil-Cre and Vil-Cre:NDI1 mice. *n* = 5 per condition (*q* = 4.6 × 10^−32^, two-sided Wald test). **g**, Schematic of the mitochondrial electron transport chain and NDI1. **h**, Succinate/fumarate ratio of jejunum in overnight-fasted mice 1 h after oral administration of vehicle or metformin (200 mg per kg body weight). *n* = 5 per condition. **i**, Jejunum citrulline levels normalized to total ion count 1 h after orally administered vehicle (water) or metformin (200 mg per kg body weight) in overnight-fasted mice. *n* = 5 per condition. **j**, Ornithine/citrulline ratio in jejunum 1 h after oral administration of vehicle (water) or metformin (200 mg per kg body weight) in overnight-fasted mice; *n* = 5 per condition. **k**, Serum citrulline levels 2.5 h after oral administration of vehicle (water) or metformin (200 mg per kg body weight) in overnight-fasted mice (normalized to thymine-D4 internal standard). Mice were also given an oral bolus of glucose (2 g per kg body weight) 30 min after vehicle/metformin administration; Vil-Cre^vehicle^ *n* = 6, Vil-Cre:NDI1^vehicle^ *n* = 7, Vil-Cre^metformin^ *n* = 9, Vil-Cre:NDI1^metformin^ *n* = 8. **l**, Liver citrulline levels normalized to total ion count 1 h after orally administered vehicle (water) or metformin (200 mg per kg body weight) in overnight-fasted mice. *n* = 5 per condition. OGTT, oral glucose tolerance test. All mice were males and 8–12 weeks of age. The vehicle was water in all experiments. For **e** and **h**–**l**, results represent the mean ± s.e.m. For **d** and **f**, results are presented as min-to-max box-and-whisker plots with whiskers as the minimum and maximum values, bounds of the box as 25th and 75th percentiles, and the centre line as the median. Statistical significance in **b** was determined by two-sided paired *t*-test. Statistical significance for **c** was determined by two-sided paired *t*-test. Statistical significance for **d** was determined by two-sided unpaired *t*-test; for **e**, one-way ANOVA with Bonferroni correction for multiple comparisons; for **h**–**l**, two-way ANOVA with Bonferroni correction for multiple comparisons. NS, not significant; *P < 0.05, **P < 0.01, ***P < 0.001, ****P < 0.0001. Panels created in BioRender: **a**, Chandel, N. https://biorender.com/wg2lxg7 (2026); **g**, Chandel, N. https://biorender.com/c15j031 (2026). Source data

**Extended Data Fig. 1:**
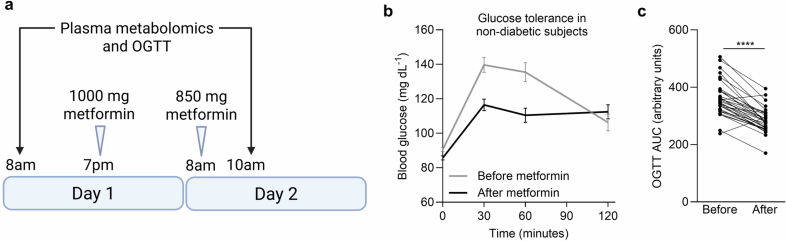
Metformin acutely improves glucose tolerance in non-diabetic humans. (**a**) Schematic illustrating sample collection and metformin dosing in the Rotroff et al. cohort (same as Fig. 1a); related to panels (**b**) and (**c**). Created in BioRender. Chandel, N. (2026) https://BioRender.com/wg2lxg7. (**b**) Glucose tolerance test in the Rotroff et al. cohort of non-diabetic subjects before and after metformin treatment. (**c**) Area under the curve of (b); n = 33. OGTT = oral glucose tolerance test. For (**b**), data are presented as mean ± SEM. Statistical significance for (**c**) was determined by two-sided paired t test. ****P < 0.0001. Source data

Postprandial glucose, which is elevated in type II diabetes, is a stronger predictor of cardiovascular disease and all-cause mortality than fasting glucose^30,31^. Metformin acutely suppresses these meal-induced glucose spikes^32,33^ and improves glucose tolerance, even in normoglycaemic individuals^34^. We confirmed the acute glucose-lowering effect of metformin by analysing publicly available data from a cohort of patients without diabetes, each of whom also underwent plasma metabolomic profiling (Extended Data Fig. 1a–c)^35^. By examining the metabolomics data, we found that citrulline was the most significantly downregulated metabolite by metformin (Fig. 1a–c and Supplementary Table 1)^35^. Similarly, in a separate cohort of patients with obesity and type II diabetes, treatment with metformin (1,500 mg per day for at least 6 months) led to a significant reduction in circulating citrulline levels compared to patients who did not receive metformin (Fig. 1d)^36^. Consistent with these findings, other groups have reported a pronounced decrease in the citrulline levels of patients with type II diabetes receiving metformin^37,38^. Interestingly, the enzyme responsible for citrulline synthesis, ornithine transcarbamylase (OTC), is localized to the mitochondrial matrix and is exclusively expressed in the liver and small intestine. While liver-produced citrulline is locally metabolized to argininosuccinate as part of the urea cycle, intestine-produced citrulline is released into the circulation to augment systemic nitric oxide synthesis^39,40^. The penultimate enzyme in the two-step citrulline synthesis pathway, mitochondrial carbamoyl phosphate synthetase I (CPS1), has the same expression pattern^41,42^ and subcellular localization as OTC and requires mitochondrial ATP, rather than ATP produced by glycolysis, to function (Extended Data Fig. 3a,b). Importantly, ATP generated in the cytosol from glycolysis does not contribute to the ATP pool in the mitochondrial matrix^43^ and, therefore, cannot compensate for reduced oxidative phosphorylation to sustain citrulline synthesis. Indeed, patients with mitochondrial dysfunction due to a mutation in *LRPPRC* display reduced plasma citrulline levels^44^. Thus, it is plausible that metformin decreases citrulline by suppressing mitochondrial activity in the small intestine.

In line with this concept, intestinal metformin levels exceed those in plasma by up to 300-fold and those in the liver by 10–100-fold, with local concentrations reaching well into the millimolar range (Fig. 1e)^8–10,45^. This is critical, as in vitro and structural studies show that metformin inhibits mitochondrial complex I only at concentrations typically achieved in the intestines under standard clinical dosing^6,7,46,47^. Mitochondrial complex I is a large (~1-MDa) protein assembly of 45 subunits and is embedded in the inner mitochondrial membrane where it supports oxidative phosphorylation and tricarboxylic acid cycle activity by donating electrons to ubiquinone, regenerating NAD^+^ and pumping protons into the inner membrane space. Mitochondrial complex I can also generate superoxide through reverse electron transport^48^. Biguanides like metformin require an intact inner mitochondrial membrane potential to accumulate in the matrix^6^ and reversibly inhibit mitochondrial complex I by binding essential residues within the ubiquinone binding channel, stabilizing the enzyme’s deactive form^47^.

Our group recently reported direct evidence that mitochondrial complex I inhibition is necessary for metformin to lower blood glucose in vivo^49^. By generating transgenic mice that ubiquitously express *Saccharomyces cerevisiae* NADH dehydrogenase (NDI1), we conferred resistance to mitochondrial complex I inhibition in all tissues of the body. NDI1 localizes to the inner mitochondrial membrane where it acts as a homodimer to catalyse ubiquinone reduction coupled to NAD^+^ regeneration, without pumping protons or generating superoxide^50,51^ (Fig. 1g). NDI1 rescues genetic complex I dysfunction and is insensitive to pharmacologic mitochondrial complex I inhibitors including biguanides^52–54^. Therefore, NDI1 can be expressed in mammalian systems to maintain electron transport chain activity in a manner that bypasses mitochondrial complex I. Mice with ubiquitous NDI1 expression were partially resistant to the glucose-lowering effect of metformin, demonstrating that complex I inhibition contributes to the antidiabetic effect of this drug^49^. Nevertheless, the specific tissue in which mitochondrial complex I is inhibited, as well as the downstream mechanism, were not identified.

**Extended Data Fig. 2:**
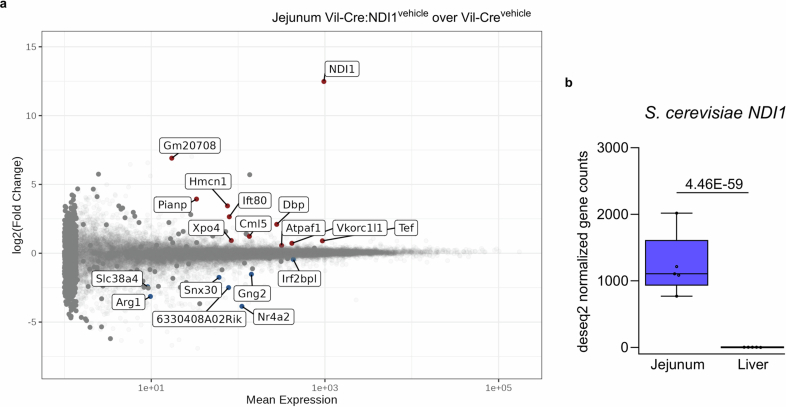
NDI1 has minimal effect on the intestinal transcriptome and is not expressed in liver of Vil-Cre:NDI1 mice. (**a**) MA plot showing differential gene expression in the jejunum of overnight fasted mice one hour after oral gavage of vehicle; Vil-Cre n = 5 and Vil-Cre:NDI1 n = 5. Genes with p-values < 0.05 are shown. (**b**) *NDI1* normalized gene expression in jejunum and liver of the same Vil-Cre:NDI1 mice one hour after oral gavage of vehicle; n = 5 (q = 4.46 ×10^−59^). Whiskers are the minimum and maximum values, bounds of the box are 25^th^ and 75^th^ percentiles, and the centre line is the median. For (**a**) and (**b**) differential expression was determined using DESeq2 with a negative binomial generalized linear model and two-sided Wald test. All mice were male, 9-12 weeks of age, and fed a standard diet. Source data

**Extended Data Fig. 3:**
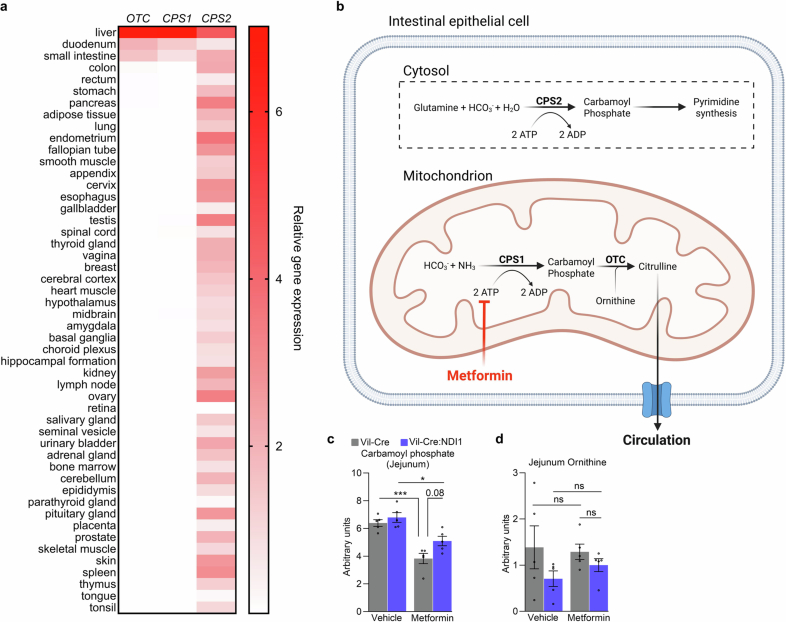
Circulating citrulline is synthesized in the mitochondria of intestinal epithelial cells. (**a**) Relative tissue-specific gene expression profiles for the enzymes that synthesize citrulline (OTC), mitochondrial carbamoyl phosphate (CPS1) and cytosolic carbamoyl phosphate (CPS2/CAD) in humans. Data retrieved 5/5/2025 from consensus RNA expression per gene based on transcriptomic profiles from The Human Protein Atlas and GTEx (https://www.proteinatlas.org/humanproteome/tissue/data#consensus_tissues_rna)^41,42^. (**b**) Schematic illustrating citrulline synthesis in the mitochondria of intestinal epithelial cells. Created in BioRender. Chandel, N. (2026) https://BioRender.com/f69n731. (**c**) Relative carbamoyl phosphate levels in jejunum one hour after oral administration of vehicle (water) or metformin (200 mg kg^−1^) in overnight-fasted mice; n = 5 per condition. (**d**) Relative ornithine levels in jejunum one hour after oral administration of vehicle (water) or metformin (200 mg kg^−1^) in overnight-fasted mice; n = 5 per condition. For (**c**) and (**d**), data are presented as mean ± SEM. Statistical significance for (**c**) and (**d**) was determined by Two-way ANOVA with Bonferroni’s correction for multiple comparisons. *P < 0.05. Source data

**Extended Data Fig. 4:**
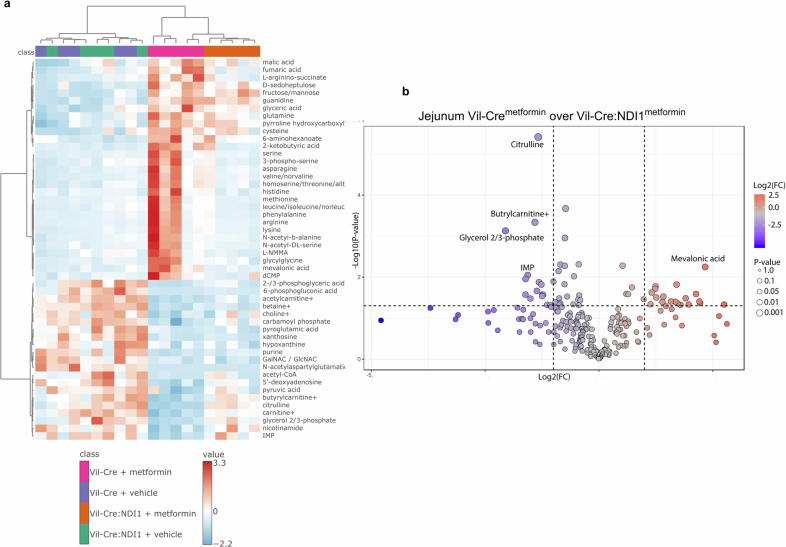
Metformin inhibits mitochondrial complex I to alter the intestinal metabolome. (**a**) Metabolomics heatmap of jejunum one hour after orally administered vehicle (water) or metformin (200 mg kg^−1^) in overnight fasted mice; n = 5 per condition. (**b**) Differentially abundant metabolites between Vil-Cre and Vil-Cre:NDI1 mice treated with metformin from (**a**). The top five metabolites are labeled; n = 5 per condition. Statistical significance was determine by two-sided unpaired t test. All mice were male, 9-12 weeks of age, and fed a standard diet.

In the present study, we generated mice that specifically express NDI1 in intestinal epithelial cells by crossing Villin-Cre mice to animals that harbour NDI1 with a lox-stop-lox sequence targeted to the *ROSA26* locus^53^ (Vil-Cre:NDI1 mice; Fig. 1f, g). RNA-sequencing analysis revealed a negligible effect of NDI1 on the intestinal transcriptome, and NDI1 was not expressed in the liver of Vil-Cre:NDI1 mice (Extended Data Fig. 2a,b). When mitochondrial complex I is inhibited, complex II (succinate dehydrogenase) becomes the primary entry point for electrons into the respiratory chain^55^. Under these conditions, forward flux through succinate dehydrogenase is elevated, resulting in the rapid consumption of succinate and production of fumarate, causing a decrease in the succinate/fumarate ratio^56^. Metformin decreases the intestinal succinate/fumarate ratio in control animals, indicating increased flux through succinate dehydrogenase. However, the succinate/fumarate ratio was unchanged by metformin in mice with intestinal NDI1 expression (Fig. 1h). Thus, metformin inhibits mitochondrial complex I in intestinal epithelium in vivo. We further assessed metformin’s effect on intestinal tissue by profiling the relative abundance of ~250 hydrophilic metabolites in the jejunum by liquid chromatography–mass spectrometry (LC–MS). Hierarchical clustering of metabolomic data showed no effect of NDI1 in vehicle-treated animals. However, NDI1 substantially diminished metformin-induced changes to the intestinal metabolome (Extended Data Fig. 4a). Differentially abundant metabolites were identified between metformin-treated animals with and without NDI1 (Supplementary Table 2). We found that citrulline showed the most significant decrease among all metabolites in response to metformin, and this reduction depended on inhibition of mitochondrial complex I (Fig. 1i and Extended Data Fig. 4b). Similarly, we observed a marked decrease in circulating citrulline levels, along with an increased ornithine/citrulline ratio in the jejunum, indicating reduced flux through OTC (Fig. 1j,k). In contrast, no change in liver citrulline was observed (Fig. 1l). Together, these data demonstrate that metformin suppresses intestinal citrulline synthesis by inhibiting mitochondrial complex I.

### Metformin inhibits mitochondrial complex I to drive intestinal glucose disposal

**Fig. 2:**
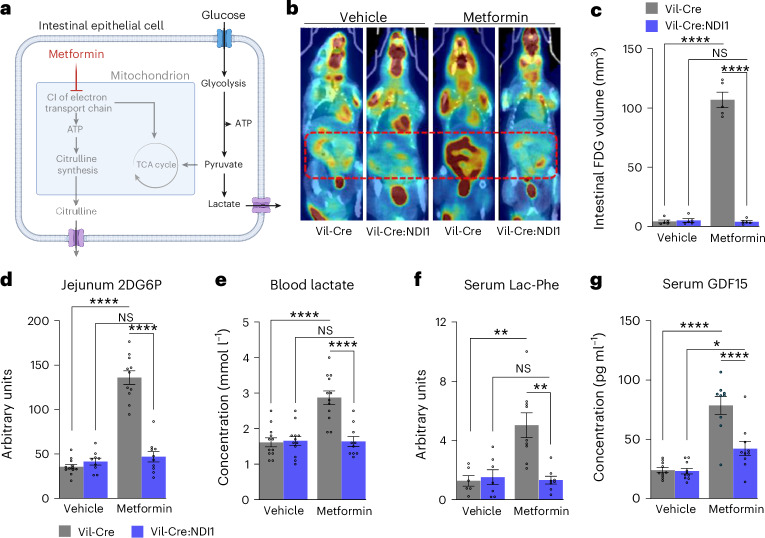
Metformin inhibits mitochondrial complex I in intestinal epithelium to drive glucose uptake and glycolysis. **a**, Model of metformin-mediated glucose disposal and suppressed citrulline synthesis in intestinal epithelium. **b**, Representative FDG-PET images of Vil-Cre and Vil-Cre:NDI1 mice with and without metformin. Mice were fasted overnight, followed by an oral gavage of vehicle or metformin (200 mg per kg body weight); 30 min later, FDG was administered via tail vein injection; PET imaging began 40 min after FDG administration and completed 60 min after FDG administration. **c**, Volume quantification of FDG signal in mice treated with vehicle (water) or 200 mg per kg body weight metformin; standard uptake value (SUV) threshold of 0.45, *n* = 5 per condition. **d**, Relative 2DG6P in the jejunum of Vil-Cre and Vil-Cre:NDI1 mice. Mice were fasted overnight, followed by an oral gavage of vehicle or metformin (200 mg per kg body weight); 30 min later, 2DG (50 mg per kg body weight) was administered in combination with 2 g per kg body weight glucose via intraperitoneal injection; 1 h later jejunum was collected to measure 2DG6P. Vil-Cre^vehicle^ *n* = 12, Vil-Cre:NDI1^vehicle^ *n* = 9, Vil-Cre^metformin^ *n* = 11, Vil-Cre:NDI1^metformin^ *n* = 10. **e**, Blood lactate levels in overnight-fasted mice. Mice were orally administered vehicle (water) or metformin (200 mg per kg body weight), followed by an oral gavage of glucose (2 g per kg body weight) 30 min later; 30 min after glucose administration, blood lactate was measured; Vil-Cre^vehicle^ *n* = 12, Vil-Cre:NDI1^vehicle^ *n* = 12, Vil-Cre^metformin^ *n* = 12, Vil-Cre:NDI1^metformin^ *n* = 10. **f**, Serum Lac-Phe levels 2.5 h after oral administration of vehicle (water) or metformin (200 mg per kg body weight) in overnight-fasted mice (normalized to thymine-D4 internal standard). Mice were also given an oral bolus of glucose (2 g per kg body weight) 30 min after vehicle/metformin administration; Vil-Cre^vehicle^ *n* = 6, Vil-Cre:NDI1^vehicle^ *n* = 7, Vil-Cre^metformin^ *n* = 9, Vil-Cre:NDI1^metformin^ *n* = 8. **g**, Serum GDF15 levels in overnight-fasted mice 8 h after oral administration of vehicle (water) or metformin (200 mg per kg body weight); Vil-Cre^vehicle^ *n* = 10, Vil-Cre:NDI1^vehicle^ *n* = 9, Vil-Cre^metformin^ *n* = 9, Vil-Cre:NDI1^metformin^ *n* = 10. All mice were males and 8–12 weeks of age. The vehicle was water for oral delivery and sterile PBS for injections. Data are presented as the mean ± s.e.m. Statistical significance in **c**–**g** was determined by two-way ANOVA with Bonferroni’s correction for multiple comparisons. **P < 0.01, ***P < 0.001, ****P < 0.0001. Panel **a** created in BioRender; Chandel, N. https://biorender.com/s90q859 (2026). Source data

**Extended Data Fig. 5:**
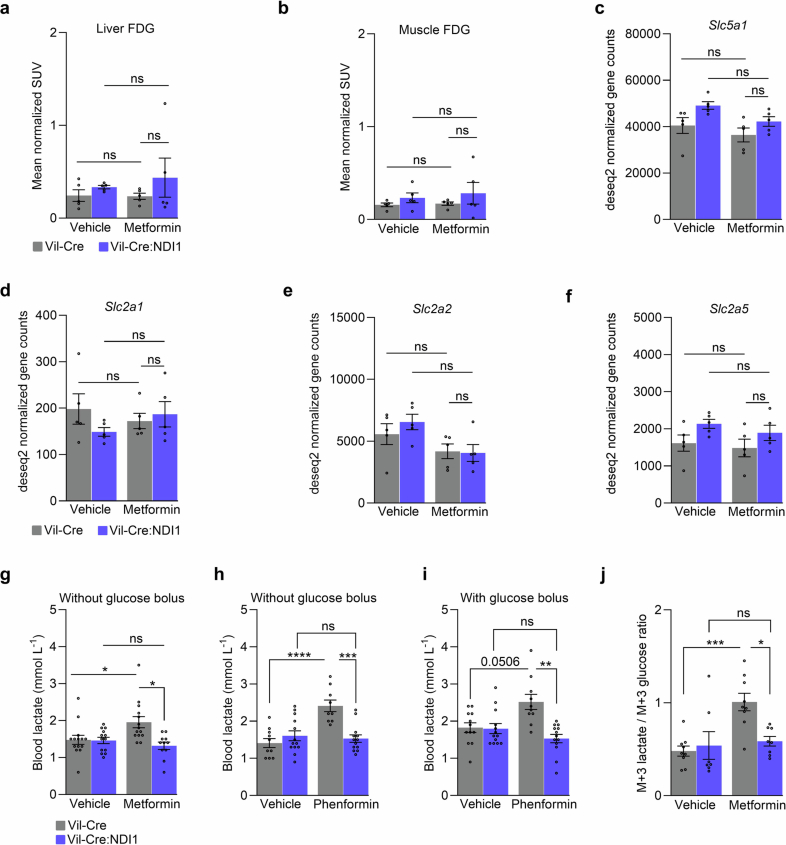
Biguanides inhibit mitochondrial complex I in intestinal epithelium to drive glycolysis. (**a**) Liver FDG accumulation; n = 5 per condition. (**b**) Hindlimb muscle FDG accumulation; n = 5 per condition. (**c**) Expression of *Slc5a1* (SGLT1) in the jejunum; n = 5 per condition. (**d**) Expression of *Slc2a1* (GLUT1) in the jejunum; n = 5 per condition. (**e**) Expression of *Slc2a2* (GLUT2) in the jejunum; n = 5 per condition. (**f**) Expression of *Slc2a5* (GLUT5) in the jejunum; n = 5 per condition. (**g**) Blood lactate one hour after oral administration of vehicle (water) or metformin (200 mg kg^−1^) to overnight-fasted mice; Vil-Cre^vehicle^ n = 14, Vil-Cre:NDI1^vehicle^ n = 13, Vil-Cre^metformin^ n = 14, Vil-Cre:NDI1^metformin^ n = 11. (**h**) Blood lactate one hour after oral administration of vehicle (water) or phenformin (100 mg kg^−1^) to overnight-fasted mice; Vil-Cre^vehicle^ n = 10, Vil-Cre:NDI1^vehicle^ n = 13, Vil-Cre^phenformin^ n = 9, Vil-Cre:NDI1^phenformin^ n = 13. (**i**) Blood lactate one hour after oral administration of vehicle (water) or phenformin (100 mg kg^−1^) to overnight-fasted mice. An oral bolus of glucose (2 g kg^−1^) was delivered 30 minutes after vehicle/phenformin administration; Vil-Cre^vehicle^ n = 12, Vil-Cre:NDI1^vehicle^ n = 13, Vil-Cre^phenformin^ n = 10, Vil-Cre:NDI1^phenformin^ n = 13. (**j**) Plasma m + 3 lactate / m + 6 glucose ratio of overnight fasted mice one hour after oral administration of vehicle (water) or metformin (200 mg kg^−1^). An oral bolus of U-^13^C_6_-glucose (2 g kg^−1^) was delivered 30 minutes after vehicle/metformin administration; Vil-Cre^vehicle^ n = 9, Vil-Cre:NDI1^vehicle^ n = 7, Vil-Cre^metformin^ n = 9, Vil-Cre:NDI1^metformin^ n = 8. All mice were male, 8-12 weeks of age, and fed a standard diet. Data are presented as mean ± SEM. Statistical significance was determined by Two-way ANOVA with Bonferroni’s correction for multiple comparisons. *P < 0.05, **P < 0.01, ***P < 0.001. Source data

Inhibition of mitochondrial complex I or other components of the electron transport chain typically results in a compensatory increase in glycolysis^57^. Indeed, an underappreciated clinical effect of metformin is enhanced intestinal glucose uptake, as measured by FDG-PET^24–26,29^. FDG is a radioactive glucose mimetic and measuring its uptake is a standard clinical cancer detection method due to the enhanced glucose uptake of tumours^58^. Discontinuation of metformin before FDG-PET scans is standard of care because metformin confounds image interpretation by enhancing intestinal FDG accumulation^28,29^. However, the underlying mechanism of this clinical effect is unknown. We hypothesized that metformin targets mitochondrial complex I to drive intestinal glucose uptake and glycolysis (Fig. 2a). To test this, we performed FDG-PET on Vil-Cre:NDI1 mice treated with metformin. Consistent with clinical observations, metformin elevates intestinal FDG uptake in control mice (Fig. 2b,c). However, in Vil-Cre:NDI1 mice, metformin fails to induce FDG uptake in the intestines (Fig. 2b,c). Importantly, metformin did not alter FDG accumulation in the liver or muscle in either genotype, indicating that its effect is specific to the intestines (Extended Data Fig. 5a,b). We also performed an orthogonal glucose uptake assay by intraperitoneally injecting mice with 2-deoxyglucose (2DG), another glucose mimetic. 2DG is taken up by cells and converted to 2-deoxyglucose-6-phosphate (2DG6P) but cannot be further metabolized. Metformin increases intestinal 2DG6P accumulation only in mice without NDI1 expression (Fig. 2d). In addition, we assessed the mRNA expression of intestinal glucose transporters and found no differences in response to metformin or NDI1 (Extended Data Fig. 5c–f). Thus, mitochondrial complex I inhibition is necessary for metformin to drive intestinal glucose uptake in a manner that does not involve the transcriptional upregulation of glucose transporters.

Lactate production is elevated in highly glycolytic cells, and metformin increases blood lactate in humans. Other biguanides, such as phenformin, were withdrawn from clinical use due to increased risk of lactic acidosis^59^. Thus, we hypothesized that metformin increases blood lactate by inhibiting mitochondrial complex I in intestinal epithelium (Fig. 2a). Consistent with this hypothesis, a single oral dose of metformin elevates blood lactate in control but not Vil-Cre:NDI1 mice (Extended Data Fig. 5g). Giving mice an oral bolus of glucose in addition to metformin further increases blood lactate in control mice. However, Vil-Cre:NDI1 mice remain insensitive to metformin-induced lactate production (Fig. 2e). Similar effects were observed with phenformin (Extended Data Fig. 5h,i). Metformin-induced glucose-to-lactate conversion was directly assessed by measuring the m + 3 lactate/m + 6 glucose ratio in blood serum after an oral dose of U-^13^C_6_-glucose. As expected, the m + 3 lactate/m + 6 glucose ratio was elevated by metformin in control but not Vil-Cre:NDI1 mice (Extended Data Fig. 5j). Together, these data demonstrate that mitochondrial complex I inhibition is required for biguanides to enhance glycolytic activity in intestinal epithelium.

**Extended Data Fig. 6:**
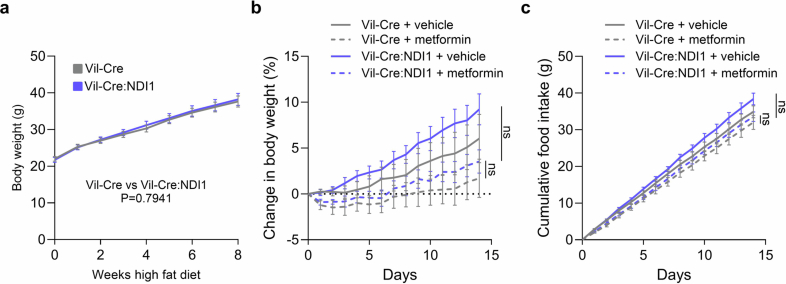
Metformin modestly slows weight gain on high-fat diet. (**a**) Weight gain of Vil-Cre and Vil-Cre: NDI1 mice on a high-fat diet (60% lard) starting at 8 weeks of age; Vil-Cre n = 12, Vil-Cre:NDI1 n = 17. (**b**) Percent body weight gain of Vil-Cre and Vil-Cre:NDI1 mice on a high-fat diet treated daily with vehicle or metformin (200 mg kg^−1^) by oral gavage; Vil-Cre^vehicle^ n = 9, Vil-Cre:NDI1^vehicle^ n = 11, Vil-Cre^metformin^ n = 9, Vil-Cre:NDI1^metformin^ n = 11. (**c**) Cumulative food intake of Vil-Cre and Vil-Cre:NDI1 mice on a high-fat diet (60% lard) treated daily with vehicle or metformin (200 mg kg^−1^) by oral gavage; Vil-Cre^vehicle^ n = 7, Vil-Cre:NDI1^vehicle^ n = 11, Vil-Cre^metformin^ n = 8, Vil-Cre:NDI1^metformin^ n = 10. All mice were male and started on high-fat diet at 8 weeks of age. Data are presented as mean ± SEM. Statistical significance was determined by two-sided unpaired t test (**a**) or Two-way ANOVA with Bonferroni’s correction for multiple comparisons (**b** and **c**) at the final time point for each experiment. Source data

Lac-Phe, a lactate derivative, and GDF15, a downstream effector of the mitochondrial integrated stress response, are also elevated by metformin in rodents and humans^60–65^. We found that Vil-Cre:NDI1 mice are refractory to metformin-induced Lac-Phe and GDF15 upregulation (Fig. 2f,g), indicating mitochondrial complex I inhibition in intestinal epithelium is necessary for metformin to elevate Lac-Phe and GDF15 levels. Metformin modestly lowers body weight in humans (~3% loss over a year)^66^. Because Lac-Phe and GDF15 have been implicated in this effect^60–65^, we assessed body weight and food intake in diet-induced obese mice treated daily with metformin (200 mg per kg body weight) for 2 weeks. We found that metformin tended to slow weight gain but did not cause weight loss (Extended Data Fig. 6b,c), consistent with previous studies in mice showing that higher doses of metformin (≥300 mg per kg body weight) are required for weight loss^60,62^.

### Intestine-specific inhibition of mitochondrial complex I is necessary for the antihyperglycaemic effect of metformin

**Fig. 3:**
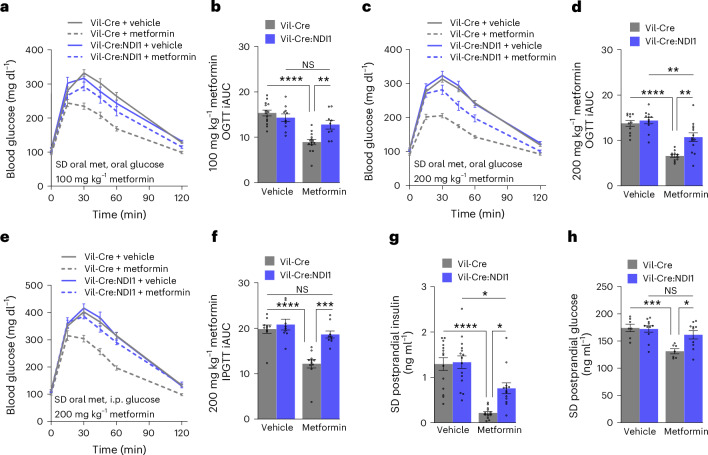
Mitochondrial complex I inhibition in intestinal epithelium is necessary for metformin to improve glycaemic control in lean mice. **a**, Oral glucose tolerance test of standard diet fed mice. Mice were fasted overnight, followed by oral administration of vehicle (water) or metformin (100 mg per kg body weight) and an oral bolus of glucose (2 g per kg body weight) 30 min later. **b**, Incremental area under the curve (iAUC; arbitrary units) of **a**; Vil-Cre^vehicle^ *n* = 14, Vil-Cre:NDI1^vehicle^ *n* = 10, Vil-Cre^metformin^ *n* = 14, Vil-Cre:NDI1^metformin^ *n* = 9. **c**, Oral glucose tolerance test of standard diet-fed mice. Vehicle (water) or metformin (200 mg per kg body weight) was orally delivered, followed by an oral bolus of glucose (2 g per kg body weight) 30 min later in overnight-fasted mice. **d**, iAUC of **c**; Vil-Cre^vehicle^ *n* = 11, Vil-Cre:NDI1^vehicle^ *n* = 11, Vil-Cre^metformin^ *n* = 11, Vil-Cre:NDI1^metformin^ *n* = 13. **e**, Glucose tolerance test in which standard diet-fed mice were overnight fasted, then orally delivered vehicle (water) or metformin (200 mg per kg body weight) and then 30 min later intraperitoneally injected with glucose (2 g per kg body weight). **f**, iAUC of **e**; Vil-Cre^vehicle^ *n* = 10, Vil-Cre:NDI1^vehicle^ *n* = 9, Vil-Cre^metformin^ *n* = 11, Vil-Cre:NDI1^metformin^ *n* = 9. **g**, Postprandial insulin levels after a fasting–refeeding assay in standard diet-fed mice; Vil-Cre^vehicle^ *n* = 14, Vil-Cre:NDI1^vehicle^ *n* = 15, Vil-Cre^metformin^ *n* = 13, Vil-Cre:NDI1^metformin^ *n* = 13. **h**, Postprandial glucose levels after a fasting–refeeding assay in standard diet-fed mice; Vil-Cre^vehicle^ *n* = 7, Vil-Cre:NDI1^vehicle^ *n* = 11, Vil-Cre^metformin^ *n* = 7, Vil-Cre:NDI1^metformin^ *n* = 10. SD, standard diet; IPGTT, intraperitoneal glucose tolerance test. All mice were male and 7–10 weeks of age. In **g** and **h**, mice were fasted overnight, followed by an oral dose of vehicle (water) or metformin (200 mg per kg body weight); 30 min later, mice were refed ad libitum for 30 min, followed by blood collection. Data are presented as the mean ± s.e.m. Statistical significance was determined by two-way ANOVA with Bonferroni’s correction for multiple comparisons. *P < 0.05, **P < 0.01, ***P < 0.001. Source data

**Fig. 4:**
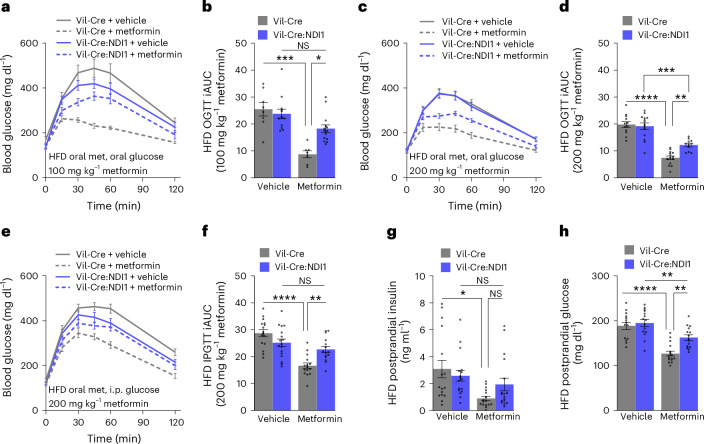
Mitochondrial complex I inhibition in intestinal epithelium is necessary for metformin to improve glycaemic control in obese mice. **a**, Oral glucose tolerance test of diet-induced obese mice fed a high-fat diet (HFD; 60% lard) for 8–10 weeks. Mice were fasted overnight, followed by oral administration of vehicle (water) or metformin (100 mg per kg body weight) and an oral bolus of glucose (2 g per kg body weight) 30 min later. **b**, iAUC of **a**; Vil-Cre^vehicle^ *n* = 8, Vil-Cre:NDI1^vehicle^ *n* = 13, Vil-Cre^metformin^ *n* = 7, Vil-Cre:NDI1^metformin^ *n* = 14. **c**, Oral glucose tolerance test of diet-induced obese mice fed a HFD for 8–10 weeks. Vehicle (water) or metformin (200 mg per kg body weight) was orally delivered, followed by an oral bolus of glucose (2 g per kg body weight) 30 min later in overnight-fasted mice. **d**, iAUC of **c**; Vil-Cre^vehicle^ *n* = 12, Vil-Cre:NDI1^vehicle^ *n* = 14, Vil-Cre^metformin^ *n* = 14, Vil-Cre:NDI1^metformin^ *n* = 11. **e**, Glucose tolerance test in which diet-induced obese mice were overnight fasted, orally delivered vehicle (water) or metformin (200 mg per kg body weight) and then 30 min later intraperitoneally injected with glucose (2 g per kg body weight). **f**, iAUC of **e**; Vil-Cre^vehicle^ *n* = 16, Vil-Cre:NDI1^vehicle^ *n* = 17, Vil-Cre^metformin^ *n* = 15, Vil-Cre:NDI1^metformin^ *n* = 15. **g**, Postprandial insulin levels after a fasting–refeeding assay in diet-induced obese mice; Vil-Cre^vehicle^ *n* = 16, Vil-Cre:NDI1^vehicle^ *n* = 17, Vil-Cre^metformin^ *n* = 16, Vil-Cre:NDI1^metformin^ *n* = 17. **h**, Postprandial glucose levels after a fasting–refeeding assay in diet-induced obese mice; Vil-Cre^vehicle^ *n* = 14, Vil-Cre:NDI1^vehicle^ *n* = 15, Vil-Cre^metformin^ *n* = 16, Vil-Cre:NDI1^metformin^ *n* = 15. All mice were started on a HFD at 8 weeks of age, and experiments were performed after 8–10 weeks of HFD feeding. In **g** and **h**, mice were fasted overnight, followed by an oral dose of vehicle (water) or metformin (200 mg per kg body weight); 30 min later, mice were refed ad libitum for 30 min, followed by blood collection. Data are presented as the mean ± s.e.m. Statistical significance was determined by two-way ANOVA with Bonferroni’s correction for multiple comparisons. *P < 0.05, **P < 0.01, ***P < 0.001. Source data

To determine whether intestinal mitochondrial complex I inhibition is necessary for the therapeutic glucose-lowering effect of metformin, we first performed glucose tolerance tests on mice fed a standard diet. Control and Vil-Cre:NDI1 mice have the same baseline glucose tolerance (Fig. 3a–f). However, the blood-glucose-lowering effect of acutely administered metformin is significantly impaired in Vil-Cre:NDI1 mice (Fig. 3a–f). This occurs independently of the glucose administration route (oral gavage or intraperitoneal injection; Fig. 3c–f) and at a low dose (100 mg per kg body weight) of metformin (Fig. 3a,b). Glucose tolerance was also assessed in diet-induced obese mice. While NDI1 did not affect body weight (Extended Data Fig. 6a), blood glucose lowering by metformin was attenuated in animals with intestinal NDI1 expression (Fig. 4a–f). Notably, resistance to metformin mediated by intestinal NDI1 was incomplete, consistent with our earlier findings in mice with ubiquitous NDI1 expression^49^, and tended to be greater at a low dose of metformin and when glucose was administered intraperitoneally rather than orally. This partial resistance suggests that metformin may engage therapeutic targets beyond mitochondrial complex I and could also reflect the inability of NDI1 to fully rescue metformin-induced suppression of complex I function.

Given these findings with acute oral administration of metformin, we next sought to determine whether the attenuation of metformin’s glucose-lowering effect by intestinal NDI1 expression persists under chronic treatment conditions. To this end, diet-induced obese mice were given metformin via drinking water for one month using an ascending dose protocol (3 mg ml⁻^1^ for 2 weeks, followed by 6 mg ml^−1^ for 2 weeks). We also assessed glycaemic control in a separate cohort of obese mice given a lower dose (1 mg ml^−1^), a concentration recently used to link PEN2 to metformin’s mechanism of action^67^.

**Extended Data Fig. 7:**
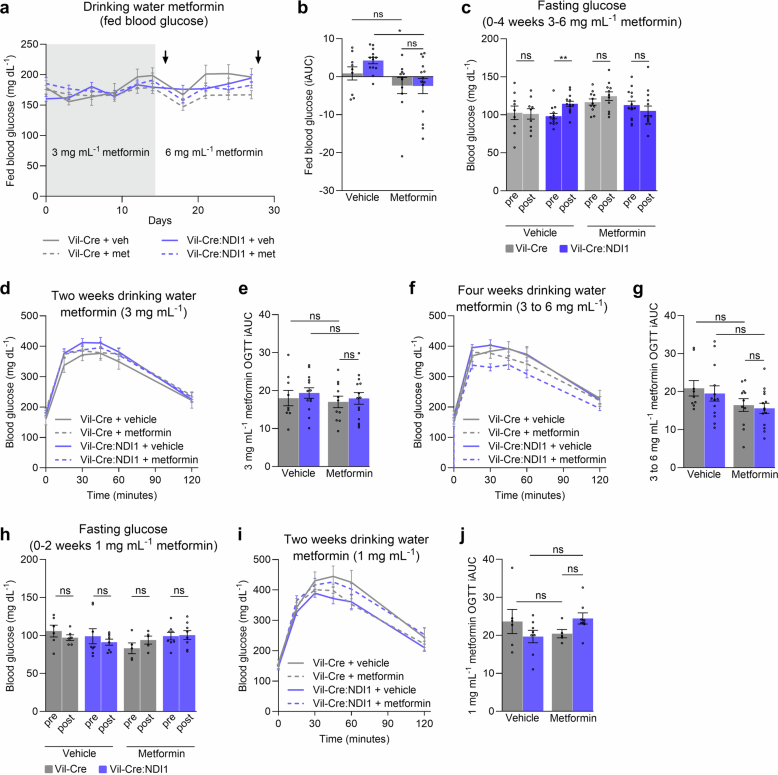
Metformin administered chronically in drinking water does not lower blood glucose. (**a**) Fed blood glucose throughout 4-week metformin treatment course. Arrows indicate when glucose tolerance tests were performed. (**b**) Incremental area under the curve of (a); Vil-Cre^vehicle^ n = 9, Vil-Cre:NDI1^vehicle^ n = 13, Vil-Cre^metformin^ n = 11, Vil-Cre:NDI1^metformin^ n = 14. (**c**) Fasting glucose before and after four weeks of metformin (3 to 6 mg mL^−1^) in drinking water; Vil-Cre^vehicle^ n = 9, Vil-Cre:NDI1^vehicle^ n = 13, Vil-Cre^metformin^ n = 11, Vil-Cre:NDI1^metformin^ n = 14. (**d**) Oral glucose tolerance test of diet-induced obese mice treated with metformin (3 mg mL⁻^1^) in drinking water for two weeks. Mice were fasted overnight, followed by oral administration of glucose (2 g kg^−1^). (**e**) Incremental area under the curve of (d); Vil-Cre^vehicle^ n = 9, Vil-Cre:NDI1^vehicle^ n = 13, Vil-Cre^metformin^ n = 11, Vil-Cre:NDI1^metformin^ n = 14. (**f**) Oral glucose tolerance test of diet-induced obese mice treated with metformin (3 to 6 mg mL⁻^1^) in drinking water for four weeks. Mice were fasted overnight, followed by oral administration of glucose (2 g kg^−1^). (**g**) Incremental area under the curve of (f); Vil-Cre^vehicle^ n = 9, Vil-Cre:NDI1^vehicle^ n = 13, Vil-Cre^metformin^ n = 11, Vil-Cre:NDI1^metformin^ n = 14. (**h**) Fasting glucose before and after two weeks of metformin (1 mg mL⁻^1^) in drinking water; Vil-Cre^vehicle^ n = 6, Vil-Cre:NDI1^vehicle^ n = 8, Vil-Cre^metformin^ n = 5, Vil-Cre:NDI1^metformin^ n = 8. (**i**) Oral glucose tolerance test of diet-induced obese mice treated with metformin (1 mg mL⁻^1^) in drinking water for two weeks. Mice were fasted overnight, followed by oral administration of glucose (2 g kg^−1^). (**j**) Incremental area under the curve of (**h**); Vil-Cre^vehicle^ n = 6, Vil-Cre:NDI1^vehicle^ n = 8, Vil-Cre^metformin^ n = 5, Vil-Cre:NDI1^metformin^ n = 8. All mice were fed a HFD for 8 weeks prior to initiation of metformin drinking water. Data are presented as mean ± SEM. Statistical significance was determined by Two-way ANOVA with Bonferroni’s correction for multiple comparisons (**b**, **e**, **g**, **j**) or repeated measures Two-way ANOVA with Bonferroni’s correction for multiple comparisons (**c**, **h**). Source data

Across all dosing regimens, metformin failed to lower fed or fasting glucose or improve glucose tolerance in either control or Vil-Cre:NDI1 mice (Extended Data Fig. 7a–j). This suggests that metformin administration via drinking water is insufficient to achieve glycaemic control. This lack of efficacy likely stems from the lower and more fluctuating metformin exposure inherent to this method, whereas acute oral gavage more closely mimics the pharmacokinetics observed in clinical settings^5^.

**Extended Data Fig. 8:**
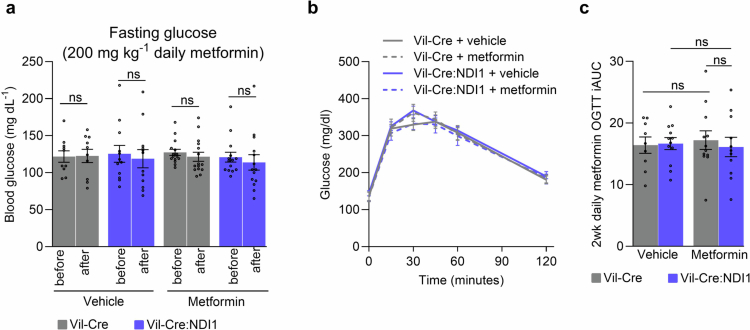
Chronic daily metformin gavage fails to lower glucose after systemic clearance. (**a**) Fasting glucose before and after two weeks of daily metformin treatment by oral gavage; Vil-Cre^vehicle^ n = 10, Vil-Cre:NDI1^vehicle^ n = 15, Vil-Cre^metformin^ n = 12, Vil-Cre:NDI1^metformin^ n = 14. (**b**) Oral glucose tolerance test following two weeks of daily metformin treatment. (**c**) Incremental area under the curve of (**b**); Vil-Cre^vehicle^ n = 10, Vil-Cre:NDI1^vehicle^ n = 15, Vil-Cre^metformin^ n = 12, Vil-Cre:NDI1^metformin^ n = 14. Statistical significance in (**a**) was determined by Two-Way repeated measures ANOVA with Bonferroni’s correction for multiple comparisons. Data are presented as mean ± SEM. Statistical significance in (**c**) was determined by Two-Way ANOVA with Bonferroni’s correction for multiple comparisons. All mice were male and started on high fat diet (60% lard) at 8 weeks of age. Experiments were performed following 13-14 weeks of high fat diet feeding. Source data

To isolate the sustained therapeutic effects of chronic treatment from the immediate effects of a single dose, we evaluated glycaemic control after allowing for a full washout of the drug. We administered a daily oral gavage of metformin (200 mg per kg body weight) to diet-induced obese mice for 2 weeks, then assessed fasting glucose and glucose tolerance 16–20 h after the final dose. Given metformin’s approximately 4-h half-life in mice^45^, this timing ensures the drug has largely cleared the system, allowing us to specifically measure the persistent physiological adaptations of chronic therapy rather than its acute pharmacologic action. We found that this dosing regimen had no effect on fasting glucose or glucose tolerance in control or Vil-Cre:NDI1 mice (Extended Data Fig. 8a–c), indicating the therapeutic glucose-lowering effect of metformin depends on repeated acute actions of the drug rather than a cumulative chronic shift in glucose homeostasis.

Having established the necessity of intestinal mitochondrial complex I inhibition for acutely administered metformin to improve glucose tolerance, we next turned to a related clinical metric of glycaemic control: postprandial glucose excursions. Postprandial hyperglycaemia is a key feature of type II diabetes and a better predictor of cardiovascular disease and all-cause mortality than fasting glucose^30,31^. Because metformin is known to suppress these meal-induced blood glucose spikes, we sought to determine whether mitochondrial complex I inhibition in intestinal epithelium is necessary for this clinical effect of metformin. To do so, we devised a simple fasting–refeeding assay in which mice were fasted overnight, treated with vehicle or metformin, and refed ad libitum 30 min before blood collection. Metformin lowered glucose and insulin levels in refed controls but this effect was attenuated in Vil-Cre:NDI1 animals (Fig. 3g,h). Similar effects were seen in diet-induced obese mice, although insulin levels were more variable (Fig. 4g,h). Thus, mitochondrial complex I inhibition in intestinal epithelium is necessary for metformin to improve postprandial glucose control.

### Intestine-specific mitochondrial complex I inhibition is essential for metformin to improve pyruvate tolerance

**Fig. 5:**
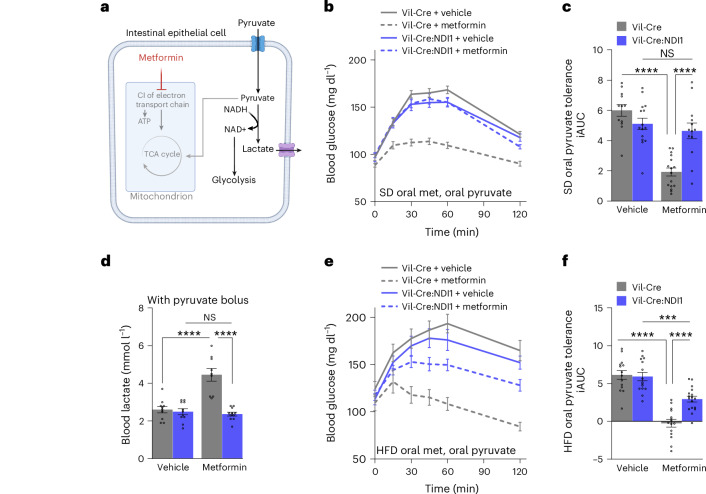
Mitochondrial complex I inhibition in intestinal epithelium is necessary for metformin to improve pyruvate tolerance. **a**, Model of metformin-induced improvement in pyruvate tolerance. **b**, Oral pyruvate tolerance test with metformin treatment on standard diet-fed mice. Mice were fasted overnight, followed by oral administration of vehicle (water) or metformin (200 mg per kg body weight) and an oral bolus of pyruvate (2 g per kg body weight) 30 min later. **c**, iAUC of **a**; Vil-Cre^vehicle^ *n* = 12, Vil-Cre:NDI1^vehicle^ *n* = 15, Vil-Cre^metformin^ *n* = 16, Vil-Cre:NDI1^metformin^ *n* = 13. **d**, Blood lactate levels in overnight-fasted mice on standard diet. Mice were orally administered vehicle (water) or metformin (200 mg per kg body weight), followed by an oral gavage of pyruvate (2 g per kg body weight) 30 min later; 30 min after pyruvate administration, blood lactate was measured; Vil-Cre^vehicle^ *n* = 10, Vil-Cre:NDI1^vehicle^ *n* = 11, Vil-Cre^metformin^ *n* = 9, Vil-Cre:NDI1^metformin^ *n* = 11. **e**, Oral pyruvate tolerance test with metformin of HFD-fed mice. Mice were fasted overnight, followed by oral administration of vehicle (water) or metformin (200 mg per kg body weight) and an oral bolus of pyruvate (2 g per kg body weight) 30 min later. **f**, iAUC of **d**; Vil-Cre^vehicle^ *n* = 16, Vil-Cre:NDI1^vehicle^ *n* = 17, Vil-Cre^metformin^ *n* = 15, Vil-Cre:NDI1^metformin^ *n* = 15. All mice were male. For standard diet-fed mice, pyruvate tolerance tests were performed on 7–10-week-old animals; blood lactate measurements were performed on 9–12-week-old animals. For HFD-fed mice, a HFD was started at 8 weeks of age and pyruvate tolerance tests were performed after 8–10 weeks of HFD feeding. Data are presented as the mean ± s.e.m. Statistical significance was determined by two-way ANOVA with Bonferroni’s correction for multiple comparisons. ***P < 0.001, ****P < 0.0001. Panel **a** created in BioRender; Chandel, N. https://biorender.com/r64q971 (2026). Source data

Historically, research on metformin has centred around its effect on hepatic gluconeogenesis, an anabolic pathway responsible for synthesizing glucose from smaller, non-carbohydrate precursors. Pyruvate tolerance tests are commonly used to assess metformin’s impact on this pathway, because pyruvate is a key substrate that the liver converts into glucose through gluconeogenesis. The resulting rise in blood glucose reflects hepatic gluconeogenesis, making the pyruvate tolerance test a useful tool for evaluating alterations in this biosynthetic process. However, pyruvate can also be metabolized in the opposite direction to form lactate and regenerate cytosolic NAD^+^ to support glycolysis during electron transport chain impairment^68^. We hypothesized that metformin could improve pyruvate tolerance by enhancing the consumption of pyruvate in the intestines (Fig. 5a).

**Extended Data Fig. 9:**
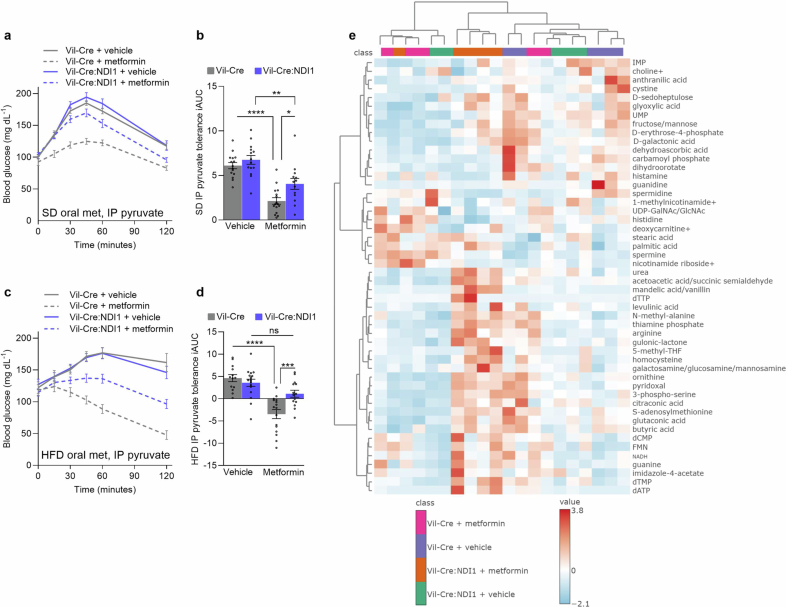
Intestinal mitochondrial complex I inhibition is necessary for metformin to improve intraperitoneal pyruvate tolerance. (**a**) Pyruvate tolerance test in which 200 mg kg^−1^ metformin was delivered by oral gavage 30 minutes before the intraperitoneal injection of pyruvate (2 g kg^−1^) in overnight-fasted mice on standard diet. (**b**) Incremental area under the curve of (**a**); Vil-Cre^vehicle^ n = 15, Vil-Cre:NDI1^vehicle^ n = 14, Vil-Cre^metformin^ n = 16, Vil-Cre:NDI1^metformin^ n = 13. (**c**) Pyruvate tolerance test in which 200 mg kg^−1^ metformin was delivered by oral gavage 30 minutes before the intraperitoneal injection of pyruvate (2 g kg^−1^) in overnight-fasted mice on high-fat diet. (**d**) Incremental area under the curve of (**c**); Vil-Cre^vehicle^ n = 13, Vil-Cre:NDI1^vehicle^ n = 17, Vil-Cre^metformin^ n = 16, Vil-Cre:NDI1^metformin^ n = 14. SD = standard diet; HFD = high-fat diet (60% lard); IP = intraperitoneal; iAUC = incremental area under the curve (arbitrary units). (**e**) Metabolomics heatmap of liver one hour after orally administered vehicle (water) or metformin (200 mg kg^−1^) in overnight fasted mice; n = 5 per condition. All mice were male. For SD-fed mice, pyruvate tolerance tests were performed on 7–10-week-old animals and the liver metabolomics was performed on 9-12-week-old animals. For HFD-fed mice, HFD was started at 8 weeks of age and pyruvate tolerance tests were performed after 8-10 weeks of HFD feeding. Data are presented as mean ± SEM. Statistical significance was determined by Two-way ANOVA with Bonferroni’s correction for multiple comparisons. *P < 0.05, **P < 0.01, ***P < 0.001. Source data

Accordingly, we found that intestinal NDI1 expression significantly attenuates metformin-induced improvement in pyruvate tolerance in both lean and obese mice (Fig. 5b,c,e,f and Extended Data Fig. 9a-d). Consistent with this, exogenous pyruvate strongly enhances metformin-induced lactate production in a manner that requires intestinal mitochondrial complex I inhibition (Fig. 5d). In contrast, metformin treatment has no effect on the liver metabolome (Extended Data Fig. 9e), suggesting that the observed improvements in pyruvate tolerance occur independently of direct hepatic changes. Together, these findings show that metformin inhibits mitochondrial complex I in intestinal epithelium to trigger a metabolic shift that redirects exogenous pyruvate away from hepatic gluconeogenesis towards the intestines to support glycolysis.

### Phenformin and berberine inhibit mitochondrial complex I in intestinal epithelium to promote glycaemic control

**Fig. 6:**
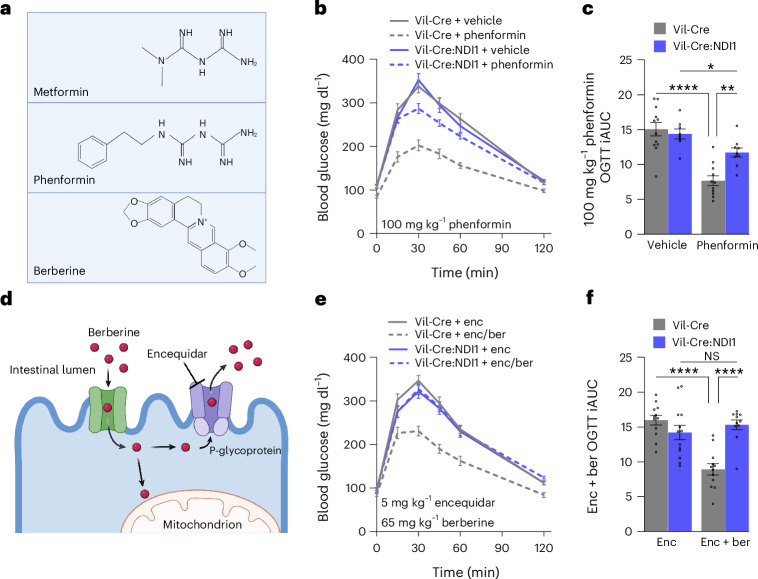
Phenformin and berberine inhibit mitochondrial complex I in intestinal epithelium to improve glucose tolerance. **a**, Chemical structures of metformin, phenformin and berberine. **b**, Oral glucose tolerance test with phenformin. Mice were fasted overnight, followed by oral administration of vehicle (water) or phenformin (100 mg per kg body weight) and an oral bolus of glucose (2 g per kg body weight) 30 min later. **c**, iAUC of **b**; Vil-Cre^vehicle^ *n* = 12, Vil-Cre:NDI1^vehicle^ *n* = 8, Vil-Cre^phenformin^ *n* = 11, Vil-Cre:NDI1^phenformin^ *n* = 9. **d**, Model of berberine’s mechanism of action in the intestinal epithelium. **e**, Oral glucose tolerance test with berberine (65 mg per kg body weight) cotreated with encequidar (5 mg per kg body weight). Mice were fasted overnight, followed by oral administration of encequidar (5 mg per kg body weight) or encequidar (5 mg per kg body weight) + berberine (65 mg per kg body weight) and an oral bolus of glucose (2 g per kg body weight) 30 min later. **f**, iAUC of **e**; Vil-Cre^enc^ *n* = 12, Vil-Cre:NDI1^enc^ *n* = 13, Vil-Cre^enc/ber^ *n* = 12, Vil-Cre:NDI1^enc/ber^ *n* = 11. All mice were male aged 7–10 (**b** and **c**) or 9–12 (**e** and **f**) weeks. Data in **c** and **f** are presented as the mean ± s.e.m. Statistical significance was determined by two-way ANOVA with Bonferroni’s correction for multiple comparisons. **P < 0.01, ****P < 0.0001. Panels created in BioRender: **a**, Chandel, N. https://biorender.com/t93z289 (2026); **d**, Chandel, N. https://biorender.com/b72n675 (2026). Source data

While metformin is currently the only FDA-approved biguanide drug, another biguanide, phenformin (Fig. 6a), was formerly used for blood glucose control before its withdrawal due to increased risk of lactic acidosis. We assessed whether intestinal mitochondrial complex I inhibition is necessary for phenformin to improve glucose tolerance. As expected, the blood-glucose-lowering effect of phenformin (100 mg per kg body weight) was attenuated in Vil-Cre:NDI1 mice (Fig. 6b,c). Thus, biguanides target mitochondrial complex I in intestinal epithelium to promote glycaemic control.

Given that biguanides originate from guanidine, a natural compound in French Lilac^69^, we wondered whether any other natural compounds selectively target mitochondrial complex I in the intestines. Putative complex I inhibitors found in nature include rotenone, annonacin and berberine, which are plant-derived allelochemicals with anti-feedant and insecticidal activities^70–72^. While rotenone and annonacin are neurotoxic to humans^70,73,74^, berberine is marketed as a dietary supplement to improve metabolic homeostasis. Clinical data are limited but suggest that berberine and metformin have overlapping pharmacological profiles, including gastrointestinal side effects^75^. Berberine, which is structurally unrelated to biguanides (Fig. 6a), is a potent mitochondrial complex I inhibitor in vitro^76^. However, its extremely low oral absorption, due to P-glycoprotein-mediated efflux into the intestinal lumen^76,77^, has made it challenging to ascertain a mechanism of action in vivo. Given berberine’s gut-restricted biodistribution, we hypothesized that it preferentially targets mitochondrial complex I in the intestinal epithelium to promote glycaemic control.

**Extended Data Fig. 10:**
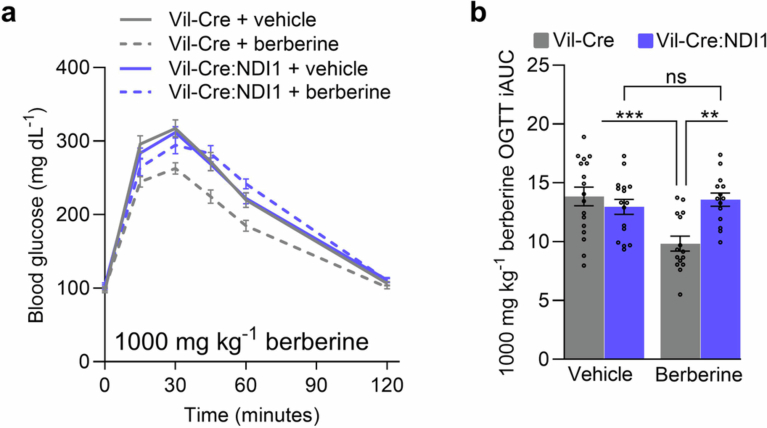
Berberine inhibits mitochondrial complex I in intestinal epithelium to improve glucose tolerance. (**a**) Oral glucose tolerance test with 1000 mg kg^−1^ berberine delivered by oral gavage 30 minutes before the oral gavage of glucose (2 g kg^−1^) in overnight-fasted mice on standard diet. (**b**) Incremental area under the curve of (c); Vil-Cre^vehicle^ n = 17, Vil-Cre:NDI1^vehicle^ n = 16, Vil-Cre^berberine^ n = 15, Vil-Cre:NDI1^berberine^ n = 14. Data are presented as mean ± SEM. All mice were male and 7-10 weeks of age. OGTT = oral glucose tolerance test, iAUC = incremental area under the curve (arbitrary units). Statistical significance was determined by Two-way ANOVA with Bonferroni’s correction for multiple comparisons. **P < 0.01. Source data

We found that a single oral dose of berberine (1,000 mg per kg body weight) improves glucose tolerance in controls but not Vil-Cre:NDI1 mice (Extended Data Fig. 10a,b), indicating intestine-selective mitochondrial complex I inhibition is necessary for berberine to lower blood glucose levels. By co-treating mice with encequidar, an intestine-specific P-glycoprotein inhibitor (Fig. 6d)^78^, we observed a far more dramatic antihyperglycaemic effect of berberine (65 mg per kg body weight) in control animals (Fig. 6e,f). Whereas, in contrast to biguanides, the glucose-lowering effect of berberine was completely blocked by intestinal NDI1 expression. These results indicate that intestine-specific mitochondrial complex I inhibition is the principal mechanism by which berberine acutely lowers blood glucose and further underscores the therapeutic utility of targeting complex I in the gut.

## Discussion

In this study, we show how metformin exerts multiple clinical effects through selective inhibition of mitochondrial complex I in the intestinal epithelium. This mechanism suppresses citrulline synthesis and drives increased glucose uptake, glycolysis and lactate production in the intestines, leading to improved glucose tolerance, pyruvate tolerance and postprandial glycaemia, along with increases in Lac-Phe and GDF15, biomarkers of mitochondrial stress that have been linked to metformin-associated body weight regulation. While intestinal complex I inhibition is necessary for these effects, the incomplete resistance to glucose lowering observed in both intestinal NDI1 mice in this study and whole-body NDI1 mice in our previous work^49^ suggests that metformin also acts through additional targets in other organs, a possibility supported by existing literature^1,5^. Alternatively, NDI1 may not fully compensate for the suppression of mammalian complex I due to its inability to pump protons or generate superoxide. Indeed, NDI1 confers modestly greater resistance when metformin is administered at low doses (Figs. 3a,b and 4a,b). In either case, our results establish that inhibition of mitochondrial complex I in intestinal epithelium is therapeutically indispensable for not only metformin, but also phenformin and the structurally unrelated molecule berberine. This shared mechanism highlights the intestines as a major site of action for these therapeutics, underscoring the clinical utility of gut-selective mitochondrial complex I inhibition in promoting glycaemic control.

Pertaining to berberine, its affinity for P-glycoproteins is consistent with its biological function as a mitochondrial toxin that defends against herbivores. P-glycoproteins are promiscuous efflux pumps that extrude potentially harmful xenobiotics into the intestinal lumen^79^. We speculate that the intestine-restricted activity of berberine is necessary for both its safety and efficacy. By limiting systemic absorption, P-glycoproteins may enable berberine to reach therapeutic levels in the gut while reducing the risk of toxicity in other tissues—a property likely to be important given that berberine is a much stronger inhibitor of mitochondrial complex I (half-maximal inhibitory concentration (IC_50_) ≈ 15 μM) compared to phenformin (IC_50_ ≈ 430 μM) and metformin (IC_50_ ≈ 19,400 μM)^46,76^.

Intestine-specific inhibition of mitochondrial complex I also accounts for metformin-induced citrulline depletion. A key question is whether the observed citrulline depletion in patients taking metformin impacts metabolic health outcomes. Circulating citrulline is the primary precursor to nitric oxide, a potent vasodilator essential for muscle perfusion during exercise^40^. Citrulline is upregulated by exercise in humans^80^ and is the dominant ingredient in many pre-workout supplements because it enhances muscle perfusion and exercise performance^81^. In contrast, metformin impairs muscle hypertrophy and aerobic capacity in response to exercise^82,83^. A direct inhibitory effect of metformin on muscle mitochondria is unlikely due to the low concentration of metformin in this tissue (Fig. 1e)^9,45^. However, it is plausible that metformin-induced citrulline depletion, and thus reduced nitric oxide production, underlies the blunted exercise benefits caused by metformin. Indeed, supplementing with citrulline could be a straightforward and scalable solution to support exercise adaptation in patients taking metformin. Should this approach be effective, it may be possible to more safely investigate the geroprotective potential of metformin^84^, enabling the rigorous clinical evaluation of its ability to delay multiple age-related diseases while mitigating harmful side effects.

Much of the literature has focused on plasma concentrations of metformin when evaluating therapeutically relevant exposures in preclinical models^85,86^. However, such an emphasis overlooks key aspects of metformin pharmacology, as plasma levels are an unreliable indicator of the drug’s distribution and accumulation in tissues^9,45^. Indeed, many rodent studies have delivered metformin via intravenous infusions and intraperitoneal injections, which bypass the gut, or have administered metformin in drinking water. While the latter approach more closely resembles clinical practice, we observed that delivering metformin this way failed to lower blood glucose (Extended Data Fig. 7).

This is likely because ad libitum access exposes animals to a variable, low-level concentration that lacks the characteristic ‘peak-and-trough’ profile seen in patients^5^. Thus, bolus dosing is essential to reproducibly achieve therapeutic efficacy. The necessity of this approach is underscored by the fact that orally delivered metformin exhibits ‘flip-flop’ pharmacokinetics, in which the rate of its appearance in plasma is slower than its rate of elimination^87^.

The ‘flip-flop’ pharmacokinetic profile of metformin arises from a functional bottleneck in the intestinal epithelium. While metformin is rapidly taken up from the lumen via multiple apical transporters (PMAT, OCTN1, OCT3, THTR-2 and SERT), its export into the bloodstream is constrained by the lone basolateral transporter, OCT1 (ref. ^88^). Unlike drinking water administration, bolus dosing creates a transient surge in the intestinal epithelium that likely overwhelms OCT1 export capacity, thereby permitting the millimolar concentrations needed for mitochondrial complex I inhibition. In contrast, the liver is exposed to roughly 1–10% of the metformin concentration achieved in the intestines^8–10^.

Accordingly, while it is generally accepted that metformin reduces endogenous glucose production through direct inhibition of hepatic gluconeogenesis, this 10- to-100-fold concentration gap challenges the primacy of the liver as the drug’s chief target. Clinical evidence shows that endogenous glucose production is not suppressed by metformin in patients with mild hyperglycaemia or early-stage type II diabetes^14–17^, who represent the vast majority of metformin users^89^. Metformin has even been found to elevate endogenous glucose production among these patient groups^15,16^. Instead, glycaemic control is achieved by increasing the rate of glucose clearance^15^. Importantly, this increase in glucose clearance is observed across diverse patient populations, ranging from normoglycaemic individuals^14,15,18,19^ to those with overt type II diabetes^22,90–93^. Therefore, enhanced glucose clearance is a robust, clinically reproducible effect of metformin.

Our findings show that metformin enhances glucose clearance by inhibiting mitochondrial complex I in the intestinal epithelium. This inhibition co-opts the intestines to function as a glucose sink, drawing in excess glucose and channelling it into glycolysis. This mechanism is essential for metformin to suppress postprandial glucose spikes. Importantly, our data suggest that metformin’s ‘chronic’ clinical benefit is not driven by a distinct steady-state mechanism but is instead the result of repeated oral bolus dosing. We demonstrate that metformin acutely improves glucose tolerance and postprandial glycaemia (Fig. 3 and 4), indicating that dosing immediately before or during meals provides a dual advantage.

While this timing is traditionally recommended to mitigate gastrointestinal side effects, it also effectively suppresses postprandial glucose excursions^32,33^. Because these spikes are stronger predictors of cardiovascular disease and all-cause mortality than fasting glucose levels^30,31^, their suppression likely accounts for metformin’s long-term survival benefits in patients with type II diabetes^94^. Consequently, our findings reinforce the clinical importance of mealtime administration to maximize these long-term metabolic and cardiovascular outcomes.

While intestine-specific mitochondrial complex I inhibition is central to metformin’s therapeutic effects, important questions remain. Our data and the work of others indicate that, in addition to intestinal complex I, metformin engages other targets, and its glucoregulatory effects vary with route of administration, tissue distribution and the stage of type II diabetes^1,4,14^. For example, the resistance conferred by NDI1 is more pronounced at lower metformin doses and when glucose is administered intraperitoneally (Figs. 3a–f and 4a–f), suggesting metformin may have distinct effects on glucose in the intestinal lumen. The gut microbiota may be particularly relevant given their direct exposure to metformin and sensitivity to host metabolism^95^. Elucidating metformin’s extra-intestinal mechanisms will also be critical to fully define its therapeutic profile and optimizing clinical use.

## Methods

### Animals

Villin-Cre mice (B6.Cg-Tg(Vil1-cre)1000Gum/J) were obtained from the Jackson Laboratory (strain no. 021504). NDI1^LSL^ mice have been described previously^53^. Homozygous Vil-Cre mice were crossed with heterozygous NDI1^LSL^ mice to generate Vil-Cre control mice and Vil-Cre:NDI1 mice. The study used littermate male Vil-Cre and Vil-Cre:NDI1 mice. Mice were housed in the Northwestern Center for Comparative Medicine vivarium in a temperature-controlled and humidity-controlled room (23 °C with 30–70% humidity range) with a 12-h light–dark cycle. Mice were monitored by research staff, as well as Northwestern Comparative Medicine animal care technicians and veterinary staff. Mice were group-housed with free access to water and standard chow (Envigo/Teklad LM-485) or a 60% lard HFD (Research diets, D12492i). Where noted in the main text and figure legends, overnight fasts were 16 h to 18 h with free access to water. After relevant procedures, mice were refed immediately. For all procedures performed on standard chow diet-fed animals, mice were 7 to 12 weeks of age. For animals fed a HFD, the HFD switch was performed at 8 weeks of age, and experiments were performed after 8 to 12 weeks of HFD feeding, unless otherwise stated in the figure legends. Metformin, phenformin, berberine and encequidar were administered via 18-gauge × 50-mm curved oral gavage needles (GavageNeedle, AFN1850C) attached to BD syringes with Luer-Lok Tips (Thermo Fisher Scientific, 14-823-30). No statistical methods were used to predetermine sample sizes, but our sample sizes are similar to those reported in previous publications^60,62^. Data collection and analysis were not performed blind to the conditions of the experiments, and no formal randomization procedure was used. The Northwestern University Institutional Animal Care and Use Committee reviewed and approved all animal procedures used in this study.

### Preparation of biguanides, berberine and encequidar

Metformin tablets (metformin hydrochloride, Granules Pharmaceuticals, NDC 70010-064-01) were crushed to a powder with a mortar and pestle and dissolved in water. Phenformin powder (Cayman Chemical, 14997) and berberine powder (Cayman Chemical, 10006427) were dissolved directly into water. Encequidar (Cayman Chemical, HM30181, 32873) was dissolved in 0.5% DMSO. For encequidar–berberine co-treatment, both drugs were dissolved in 0.5% DMSO.

### Tissue quantification of metformin

Five male C57BL/6J mice (10 weeks old) were fasted overnight (16–18 h), followed by an oral gavage of metformin (200 mg per kg body weight). One hour later, blood was collected via tail vein nick into microhaematocrit tubes (Thermo Fisher Scientific, 22-362-566), and mice were euthanized. The intestine (jejunum), muscle (soleus) and liver tissues were collected and frozen on dry ice. Blood was centrifuged in 1.5-ml microcentrifuge tubes at 10,000*g* at 4 °C for 10 min to collect plasma. Plasma and tissue were stored at −80 °C until further processing. Tissue was homogenized using a QIAGEN TissueRuptor II in cold acetonitrile–water (80:20, vol/vol) with a 2 μM metformin-D6 (Cayman Chemical, no. 16921) internal standard (80 μl acetonitrile–water per 1 mg tissue). A total of 10 μl of plasma was added to 90 μl acetonitrile–water (80:20, vol/vol). The samples underwent three freeze–thaw cycles, then were centrifuged at 17,000*g* at 4 °C for 10 min. Supernatants containing metformin and soluble metabolites were collected.

As previously described^49^, a standard curve was made using metformin concentrations ranging from 25 nM to 200 μM (Cayman Chemical, 13118) in 80:20 acetonitrile–water spiked with 2 uM metformin-D6 (Cayman Chemical, 16921). High-performance liquid chromatography and triple quadrupole tandem mass spectrometry (HPLC–MS/MS) analysed the standards and samples. The system consists of a TSQ (Thermo Fisher Scientific) in line with an electrospray ion source (ESI) and Vanquish (Thermo Fisher Scientific) ultra-high-performance liquid chromatography system with a binary pump, degasser and auto-sampler outfitted with an XBridge C18 column (Waters, dimensions of 2.1 mm by 50 mm, 3.5 μM). Solute separation was achieved through isocratic elution with the mobile phase containing 0.1% formic acid in acetonitrile–water (65:35, vol/vol) at 0.15 ml min^−1^. The capillary ESI was set to 300 °C in positive mode, with sheath gas at 35 arbitrary units, auxiliary gas at 5 arbitrary units and the spray voltage at 3.5 kV. Selective reaction monitoring of the protonated precursor ion and the related product ions for metformin and metformin-D6 (mass/charge ratio (*m/z*) 130.15 → 71, 136.15 → 77, respectively) was performed. The standard curve was calculated from the peak area ratio of targets to internal standard with a linear regression *R*^2^ = 0.99998. Data were acquired using Xcalibur 4.1 software and analysed via TraceFinder 4.1 (Thermo Fisher Scientific). Tissue and plasma metformin concentrations were calculated using the standard curve after normalizing to the internal metformin-D6 standard.

### Glucose and pyruvate tolerance tests and blood lactate quantification

For glucose and pyruvate tolerance tests, mice were fasted overnight (16–18 h) followed by an oral gavage of vehicle or drug (that is, metformin, phenformin, berberine or encequidar + berberine). Thirty minutes later, fasting blood glucose was measured via a tail vein nick with a Contour Next glucose test meter. Immediately after the blood glucose measurement, mice were given an oral gavage or intraperitoneal injection of glucose or pyruvate (2 g per kg body weight). For oral dosing, glucose/pyruvate was dissolved in water; for intraperitoneal dosing, glucose/pyruvate was dissolved in sterile PBS. Blood glucose was measured 15 min, 30 min, 45 min, 60 min and 120 min following glucose/pyruvate administration. Glucose tolerance tests were performed the same way after 2 weeks of metformin treatment in drinking water, except that mice did not receive an acute dose of metformin on the test day. The iAUC was calculated using GraphPad Prism software v10.4.1.

Similarly, mice were fasted overnight (16–18 h) for blood lactate measurements followed by oral gavage of vehicle or drug (that is, metformin or phenformin). Thirty minutes later, mice were given an oral bolus of water or glucose/pyruvate (2 g per kg body weight). After an additional 30 min, blood lactate was determined via a tail vein nick with a Nova Biomedical Lactate Plus meter.

### Body weight and food intake measurements

Mice were placed on a HFD beginning at 8 weeks of age. After 4 weeks on the diet, they were single-housed for 3 days before undergoing a 4-day oral gavage conditioning period, during which they received one daily gavage of water. Following conditioning, mice and their food intake were measured daily for 2 weeks. During this monitoring period, mice received a daily oral gavage of either vehicle (water) or metformin (200 mg per kg body weight).

### Fasting–refeeding assay

Mice were fasted overnight (16–18 h), followed by single-housing and an oral gavage of vehicle (water) or metformin (200 mg per kg body weight). Thirty minutes later, mice were refed ad libitum for half an hour. After refeeding, blood glucose was measured via a tail vein nick with a Contour Next blood glucose meter. For insulin measurement, additional blood was collected into microhaematocrit tubes (Thermo Fisher Scientific, 22-362-566). Following blood collection, mice were returned to their original cages. Blood within microhematocrit tubes was transferred to 1.5-ml microcentrifuge tubes on ice and then spun down at 10,000*g* at 4 °C for 10 min. Plasma was collected and stored at −80 °C until insulin was quantified using the Ultra-Sensitive Mouse Insulin ELISA kit (Crystal Chem, 90080) according to the manufacturer’s instructions.

### Plasma GDF15 quantification

Mice were fasted overnight (16–18 h), followed by an oral gavage of vehicle (water) or metformin (200 mg per kg body weight). Eight hours later, blood was collected, and plasma was acquired/stored as in the fasting–refeeding assay above. GDF15 was measured using the Mouse/Rat GDF15 Quantikine ELISA kit (MDG150) according to the manufacturer’s instructions.

### Serum quantification of Lac-Phe and citrulline

Mice were fasted overnight (16–18 h), followed by an oral gavage of vehicle (water) or metformin (200 mg per kg body weight). Thirty minutes later, mice were given an oral glucose bolus (2 g per kg body weight). Blood was collected 2 h after the glucose bolus, and plasma was acquired/stored as above. Five microlitres of plasma was added to 170 µl of cold 100% HPLC-grade methanol to extract metabolites. Samples were vortexed and then incubated on dry ice for 5 min. Samples were then centrifuged for 10 min at 16,000*g* at 4 °C. Following centrifugation, 50 µl of supernatant was added to 50 µl of cold methanol/water (80:20, vol/vol) with a thymine-D4 internal standard at 200 ng ml^−1^. Samples were then vortexed and centrifuged for 10 min at 16,000*g* at 4 °C. One-hundred microlitres of supernatant was transferred to HPLC tubes for metabolite quantification as previously reported^96^. The supernatant was collected for LC–MS analysis. LC flow injection analysis was performed on an Xbridge BEH amide HILIC column (Waters) with the UltiMate 3000 HPLC system (Thermo Fisher). Solvent A was 95:5 water:acetonitrile with 20 mM ammonium acetate and 20 mM ammonium hydroxide at pH 9.4. Solvent B was acetonitrile. The gradient used for metabolite separation was: 0 min, 90% B; 2 min, 90% B; 3 min, 75% B; 7 min, 75% B; 8 min, 70% B; 9 min, 70% B; 10 min, 50% B; 12 min, 50% B; 13 min, 25% B; 14 min, 25% B; 16 min, 0% B; 21 min, 0% B; 21 min, 90% B; and 25 min, 90% B. MS analysis was performed on an Exploris 240 mass spectrometer (Thermo Fisher) in polarity switching mode, scanning an *m*/*z* range from 70 to 1,000. Data were analysed using El-MAVEN Software (Elucidata; elucidata.io)^97^.

### Metabolomics and plasma U-^13^C_6_-glucose tracing

Mice were fasted overnight (16–18 h), followed by an oral gavage of vehicle (water) or metformin (200 mg per kg body weight). One hour later, mice were euthanized with isoflurane, and jejunum and liver were collected. Before freezing on dry ice, luminal contents of jejunum were removed by flushing with PBS and applying gentle pressure. Samples were stored at −80 °C until further processing. Jejunum and liver were mechanically homogenized in 20 μl cold acetonitrile–water (80:20, vol/vol) per 1 mg tissue and samples were frozen and thawed three times before spinning down at 10,000*g* for 10 min at 4 °C. Supernatants containing soluble metabolites were collected, and HPLC–MS/MS was performed. Metabolite separation was achieved through gradient elution, and data were acquired using Xcaliber software (v4.1 Thermo Fisher Scientific). Data analysis was performed using MetaboAnalyst (v5.0)^98^. Metabolite abundance was normalized to the total ion count for each sample. Hierarchical clustering (Ward) was performed with the top 50 differentially abundant metabolites (analysis of variance (ANOVA)) and shown as a heat map.

For U-^13^C_6_-glucose tracing, overnight-fasted mice were given an oral gavage of vehicle (water) or metformin (200 mg per kg body weight) followed by an oral dose of U-^13^C_6_-glucose (2 g per kg body weight) 30 min later. Thirty minutes after the U-^13^C_6_-glucose administration, blood was collected via a tail vein nick and plasma acquired/stored as above. Plasma was diluted 1:100 (vol/vol) in cold acetonitrile–water (80:20, vol/vol) and freeze/thaw cycles were performed three times before centrifugation at 10,000*g* for 10 min at 4 °C. Supernatants were collected and HPLC–MS/MS was performed. The m + 3 lactate/m + 6 glucose ratio was calculated on a per-sample basis with ion counts.

### Human citrulline and metabolomic analysis

Publicly available human metabolomics data were obtained from Rotroff et al.^35^ and Aleidi et al.^36^, both released under a Creative Commons licence CC BY 4.0. For Rotroff et al., citrulline levels from time point A (before metformin) and time point C (after metformin) were obtained from supplementary data sheet 4 of the original manuscript. The volcano plot was generated using metabolite levels from supplementary data sheet 4 with a custom R script (available on Zenodo); all unknown metabolites were removed before analysis. Glucose tolerance data from Rotroff et al. were obtained from supplementary data sheet 3 of the original manuscript. Relative citrulline levels from Aleidi et al. were obtained from supplementary data sheet 3 of the original manuscript. Notably, the original data from Aleidi et al. are presented in technical duplicates. We averaged these values and present only biological replicates.

### RNA sequencing

Mice were fasted overnight (16–18 h), followed by an oral gavage of vehicle (water). One hour later, they were euthanized with isoflurane, and the jejunum was collected. Luminal contents were removed by flushing with PBS and applying gentle pressure, and jejunal tissue was flash-frozen on dry ice. Tissue samples were stored at −80 °C until RNA was extracted according to the Zymo Research Direct-zol RNA MiniPrep instructions with TriReagent kit (R2051-A).

RNA sequencing was performed as previously descirbed^49^. Briefly, RNA was quantified, and its quality was determined using an Agilent 4200 TapeStation with the RNA ScreenTape System (Agilent Technologies). RNA libraries were prepared using the NEBNext Ultra DNA Library Prep Kit for Illumina (NEB E7370L). Library quality was assessed using the TapeStation 4200 High Sensitivity DNA tapes (Agilent Technologies). Dual-indexed libraries were pooled, and single-end sequenced with an Illumina NextSeq2000 instrument for 100 cycles. BCL Convert v1.2.0 was used to generate FASTQ files. Samples were processed via the publicly available nf-core/rnaseq pipeline v3.12.0 implemented in Nextflow v23.04.3 using Singularity v3.8.1. The minimal command used was: nextflow run nf-core/rnaseq -r ‘3.12.0’ -profile nu_genomics --genome ‘GRCm38’ --additional_fasta ‘S288C_YML120C_NDI1_genomic.fasta’ --star_index false. Reads were trimmed with trimGalore! v0.6.7 and aligned to the hybrid genome incorporating the NDI1 sequence with STAR v2.6.1d. Gene-level assignments were made with Salmon v1.10.1.

Data analysis was performed using custom scripts in R v4.4.0 with DESeq2 v1.46.0. A local gene dispersion model and Wald tests were used for pairwise comparisons. An alpha threshold of 0.05 was applied for differential expression analysis.

### ^18^F-FDG-PET/CT

Mice were fasted overnight (16–18 h), followed by an oral gavage of vehicle (water) or metformin (200 mg per kg body weight). Thirty minutes later, mice were injected via the tail vein with ^18^F-FDG (~10.5 MBq; Sofie Biosciences). Following 40 min of awake incubation, mice were anaesthetized with 1–3% isoflurane and imaged by PET and computed tomography (CT) on the NanoScan8 PET/CT system (Mediso). Images were analysed using ITK-SNAP (3.0) software. SUVs were internally normalized to the brain, and intestinal FDG accumulation was determined by calculating intestinal volume with SUV > 0.45.

### 2DG uptake assay

Mice were fasted overnight (16–18 h), followed by an oral gavage of vehicle (water) or metformin (200 mg per kg body weight). Thirty minutes later, mice were intraperitoneally injected with glucose (2 g per kg body weight) and 2DG (50 mg per kg body weight). One hour later, mice were euthanized, and the jejunum was collected. Before freezing on dry ice, luminal contents were removed by flushing with PBS and applying gentle pressure. Samples were stored at −80 °C until further processing. The following procedure was adapted from the Promega Glucose Uptake-Glo kit (J1342). Jejunum was mechanically homogenized in 10 μl Stop Buffer per mg tissue. Following homogenization, an equal volume of Neutralization Buffer was added and the samples were vortexed. Samples were then frozen and thawed once and centrifuged for 5 min at 10,000*g* to pellet cell debris. Supernatant was transferred to a fresh microcentrifuge tube and diluted at a 1:10 ratio in PBS. Relative 2DG6P levels were determined by following the manufacturer’s instructions.

### Statistical analysis

Except for metabolomics and RNA sequencing, data analysis and visualization were performed in GraphPad Prism v10.4.1. All data points are biological replicates. The statistical methods for each experiment are described in the figure legends. Data distribution was assumed to be normal, but this was not formally tested. In Fig. 4g in the Vil-Cre^metformin^ group, there was a single data point that was below zero. Because this is not a biologically possible insulin concentration, this data point was excluded from analysis.

### Reporting summary

Further information on research design is available in the Nature Portfolio Reporting Summary linked to this article.

## Supplementary information

Supplementary Information Supplementary Tables 1 and 2.

Reporting Summary

## Source data

Source Data Fig. 1 Statistical source data.

Source Data Fig. 2 Statistical source data.

Source Data Fig. 3 Statistical source data.

Source Data Fig. 4 Statistical source data.

Source Data Fig. 5 Statistical source data.

Source Data Fig. 6 Statistical source data.

Source Data Extended Data Fig./Table 1 Statistical source data.

Source Data Extended Data Fig./Table 2 Statistical source data.

Source Data Extended Data Fig./Table 3 Statistical source data.

Source Data Extended Data Fig./Table 5 Statistical source data.

Source Data Extended Data Fig./Table 6 Statistical source data.

Source Data Extended Data Fig./Table 7 Statistical source data.

Source Data Extended Data Fig./Table 8 Statistical source data.

Source Data Extended Data Fig./Table 9 Statistical source data.

Source Data Extended Data Fig./Table 10 Statistical source data.

## Data Availability

The paper contains all the data required to evaluate its conclusions. Transcriptomic data have been deposited to the Gene Expression Omnibus under accession GSE293164. Metabolomic data have been deposited to the Metabolomics Workbench (study ID: ST003841). Source data are provided with this paper.

## Code availability

Code used for human metabolomic and mouse RNA-sequencing analyses is available on Zenodo (10.5281/zenodo.15396719)^99^.

## References

[1] Foretz, M., Guigas, B. & Viollet, B. Metformin: update on mechanisms of action and repurposing potential. *Nat. Rev. Endocrinol.* **19**, 460–476 (2023). 37130947 10.1038/s41574-023-00833-4 PMC10153049

[2] Bailey, C. J. Biguanides and NIDDM. *Diabetes Care* **15**, 755–772 (1992). 1600835 10.2337/diacare.15.6.755

[3] DeFronzo, R. A., Barzilai, N. & Simonson, D. C. Mechanism of metformin action in obese and lean noninsulin-dependent diabetic subjects. *J. Clin. Endocrinol. Metab.* **73**, 1294–1301 (1991). 1955512 10.1210/jcem-73-6-1294

[4] Hundal, R. S. et al. Mechanism by which metformin reduces glucose production in type 2 diabetes. *Diabetes* **49**, 2063–2069 (2000). 11118008 10.2337/diabetes.49.12.2063 PMC2995498

[5] LaMoia, T. E. & Shulman, G. I. Cellular and molecular mechanisms of metformin action. *Endocr. Rev.* **42**, 77–96 (2021). 32897388 10.1210/endrev/bnaa023 PMC7846086

[6] Owen, M. R., Doran, E. & Halestrap, A. P. Evidence that metformin exerts its anti-diabetic effects through inhibition of complex 1 of the mitochondrial respiratory chain. *Biochem. J.* **348**, 607–614 (2000). 10839993 PMC1221104

[7] El-Mir, M.-Y. et al. Dimethylbiguanide inhibits cell respiration via an indirect effect targeted on the respiratory chain complex I. *J. Biol. Chem.* **275**, 223–228 (2000). 10617608 10.1074/jbc.275.1.223

[8] Bailey, C. J., Wilcock, C. & Scarpello, J. H. B. Metformin and the intestine. *Diabetologia* **51**, 1552–1553 (2008). 18528677 10.1007/s00125-008-1053-5

[9] Gormsen, L. C. et al. In vivo imaging of human 11C-metformin in peripheral organs: dosimetry, biodistribution, and kinetic analyses. *J. Nucl. Med.* **57**, 1920–1926 (2016). 27469359 10.2967/jnumed.116.177774

[10] Wilcock, C. & Bailey, C. Accumulation of metformin by tissues of the normal and diabetic mouse. *Xenobiotica* **24**, 49–57 (1994). 8165821 10.3109/00498259409043220

[11] Madiraju, A. K. et al. Metformin suppresses gluconeogenesis by inhibiting mitochondrial glycerophosphate dehydrogenase. *Nature* **510**, 542–546 (2014). 24847880 10.1038/nature13270 PMC4074244

[12] Xie, D. et al. Let-7 underlies metformin-induced inhibition of hepatic glucose production. *Proc. Natl Acad. Sci. USA* **119**, e2122217119 (2022). 35344434 10.1073/pnas.2122217119 PMC9169108

[13] LaMoia, T. E. et al. Metformin, phenformin, and galegine inhibit complex IV activity and reduce glycerol-derived gluconeogenesis. *Proc. Natl Acad. Sci. USA* **119**, e2122287119 (2022). 35238637 10.1073/pnas.2122287119 PMC8916010

[14] Sarabhai, T. et al. 222-OR: metformin reduces fasting glycemia in well-controlled type 2 diabetes by promoting aerobic glycolysis independent of decreasing endogenous glucose production. *Diabetes*10.2337/db23-222-OR (2023).

[15] Gormsen, L. C. et al. Metformin increases endogenous glucose production in non-diabetic individuals and individuals with recent-onset type 2 diabetes. *Diabetologia* **62**, 1251–1256 (2019). 30976851 10.1007/s00125-019-4872-7

[16] Konopka, A. R. et al. Hyperglucagonemia mitigates the effect of metformin on glucose production in prediabetes. *Cell Rep.* **15**, 1394–1400 (2016). 27160898 10.1016/j.celrep.2016.04.024 PMC4871720

[17] Dietsche, K. B. et al. Glycemia and gluconeogenesis with metformin and liraglutide: a randomized trial in youth-onset type 2 diabetes. *J. Clin. Endocrinol. Metab.* **109**, 1361–1370 (2024). 37967247 10.1210/clinem/dgad669 PMC11031226

[18] Christensen, M. M. H. et al. Endogenous glucose production increases in response to metformin treatment in the glycogen-depleted state in humans: a randomised trial. *Diabetologia* **58**, 2494–2502 (2015). 26271344 10.1007/s00125-015-3733-2

[19] McCreight, L. J. et al. Metformin increases fasting glucose clearance and endogenous glucose production in non-diabetic individuals. *Diabetologia* **63**, 444–447 (2020). 31758212 10.1007/s00125-019-05042-1 PMC6946719

[20] Widén, E. I., Eriksson, J. G. & Groop, L. C. Metformin normalizes nonoxidative glucose metabolism in insulin-resistant normoglycemic first-degree relatives of patients with NIDDM. *Diabetes* **41**, 354–358 (1992). 1551495 10.2337/diab.41.3.354

[21] Rittig, N. et al. Metformin stimulates intestinal glycolysis and lactate release: a single-dose study of metformin in patients with intrahepatic portosystemic stent. *Clin. Pharmacol. Ther.* **110**, 1329–1336 (2021). 34331316 10.1002/cpt.2382

[22] Féry, F., Plat, L. & Balasse, E. O. Effects of metformin on the pathways of glucose utilization after oral glucose in non-insulin-dependent diabetes mellitus patients. *Metabolism* **46**, 227–233 (1997). 9030834 10.1016/s0026-0495(97)90307-3

[23] Johnson, A. et al. The impact of metformin therapy on hepatic glucose production and skeletal muscle glycogen synthase activity in overweight type II diabetic patients. *Metabolism* **42**, 1217–1222 (1993). 8412779 10.1016/0026-0495(93)90284-u

[24] Gontier, E. et al. High and typical 18 F-FDG bowel uptake in patients treated with metformin. *Eur. J. Nucl. Med. Mol. Imaging* **35**, 95–99 (2008). 17786437 10.1007/s00259-007-0563-6

[25] Bybel, B., Greenberg, I. D., Paterson, J., Ducharme, J. & Leslie, W. D. Increased F-18 FDG intestinal uptake in diabetic patients on metformin: a matched case–control analysis. *Clin. Nucl. Med.* **36**, 452–456 (2011). 21552023 10.1097/RLU.0b013e318217399e

[26] Koffert, J. P. et al. Metformin treatment significantly enhances intestinal glucose uptake in patients with type 2 diabetes: results from a randomized clinical trial. *Diabetes Res. Clin. Pract.* **131**, 208–216 (2017). 28778047 10.1016/j.diabres.2017.07.015

[27] Tobar, N. et al. Metformin acts in the gut and induces gut-liver crosstalk. *Proc. Natl Acad. Sci. USA* **120**, e2211933120 (2023). 36656866 10.1073/pnas.2211933120 PMC9942892

[28] Surasi, D. S., Bhambhvani, P., Baldwin, J. A., Almodovar, S. E. & O’Malley, J. P. ^18^F-FDG PET and PET/CT patient preparation: a review of the literature. *J. Nucl. Med. Technol.* **42**, 5–13 (2014). 24503347 10.2967/jnmt.113.132621

[29] Oh, J.-R. et al. Impact of medication discontinuation on increased intestinal FDG accumulation in diabetic patients treated with metformin. *AJR Am. J. Roentgenol.* **195**, 1404–1410 (2010). 10.2214/AJR.10.4663 21098202

[30] Group, D. S. & Group, E. D. E. Glucose tolerance and cardiovascular mortality: comparison of fasting and 2-hour diagnostic criteria. *Arch. Intern. Med.* **161**, 397–405 (2001). 11176766 10.1001/archinte.161.3.397

[31] Cavalot, F. et al. Postprandial blood glucose predicts cardiovascular events and all-cause mortality in type 2 diabetes in a 14-year follow-up: lessons from the San Luigi Gonzaga Diabetes Study. *Diabetes Care* **34**, 2237–2243 (2011). 21949221 10.2337/dc10-2414 PMC3177732

[32] Lund, S. S. et al. Impact of metformin versus the prandial insulin secretagogue, repaglinide, on fasting and postprandial glucose and lipid responses in non-obese patients with type 2 diabetes. *Eur. J. Endocrinol.* **158**, 35–46 (2008). 18166815 10.1530/EJE-07-0500

[33] Bahne, E. et al. Metformin-induced glucagon-like peptide-1 secretion contributes to the actions of metformin in type 2 diabetes. *JCI Insight* **3**, e93936 (2018). 30518693 10.1172/jci.insight.93936 PMC6328020

[34] Stocker, S. L. et al. The effect of novel promoter variants in MATE1 and MATE2 on the pharmacokinetics and pharmacodynamics of metformin. *Clin. Pharmacol. Ther.* **93**, 186–194 (2013). 23267855 10.1038/clpt.2012.210 PMC3671611

[35] Rotroff, D. M. et al. Pharmacometabolomic assessment of metformin in non-diabetic, African Americans. *Front. Pharmacol.* **7**, 135 (2016). 27378919 10.3389/fphar.2016.00135 PMC4906013

[36] Aleidi, S. M. et al. Obesity connected metabolic changes in type 2 diabetic patients treated with metformin. *Front. Pharmacol.* **11**, 616157 (2021). 33664666 10.3389/fphar.2020.616157 PMC7921791

[37] Breier, M. et al. Immediate reduction of serum citrulline but no change of steroid profile after initiation of metformin in individuals with type 2 diabetes. *J. Steroid Biochem. Mol. Biol.* **174**, 114–119 (2017). 28801099 10.1016/j.jsbmb.2017.08.004

[38] Adam, J. et al. Metformin effect on nontargeted metabolite profiles in patients with type 2 diabetes and in multiple murine tissues. *Diabetes* **65**, 3776–3785 (2016). 27621107 10.2337/db16-0512

[39] Curis, E. et al. Almost all about citrulline in mammals. *Amino Acids* **29**, 177–205 (2005). 16082501 10.1007/s00726-005-0235-4

[40] Bahadoran, Z., Mirmiran, P., Kashfi, K. & Ghasemi, A. Endogenous flux of nitric oxide: citrulline is preferred to Arginine. *Acta Physiol.* **231**, e13572 (2021). 10.1111/apha.13572 33089645

[41] Consortium, G. et al. The Genotype-Tissue Expression (GTEx) pilot analysis: multitissue gene regulation in humans. *Science* **348**, 648–660 (2015). 25954001 10.1126/science.1262110 PMC4547484

[42] Uhlén, M. et al. Tissue-based map of the human proteome. *Science* **347**, 1260419 (2015). 25613900 10.1126/science.1260419

[43] Imamura, H. et al. Visualization of ATP levels inside single living cells with fluorescence resonance energy transfer-based genetically encoded indicators. *Proc. Natl Acad. Sci. USA* **106**, 15651–15656 (2009). 19720993 10.1073/pnas.0904764106 PMC2735558

[44] Legault, J. T. et al. A metabolic signature of mitochondrial dysfunction revealed through a monogenic form of Leigh syndrome. *Cell Rep.* **13**, 981–989 (2015). 26565911 10.1016/j.celrep.2015.09.054 PMC4644511

[45] Zake, D. M. et al. Physiologically based metformin pharmacokinetics model of mice and scale-up to humans for the estimation of concentrations in various tissues. *PLoS ONE* **16**, e0249594 (2021). 33826656 10.1371/journal.pone.0249594 PMC8026019

[46] Bridges, H. R., Jones, A. J. Y., Pollak, M. N. & Hirst, J. Effects of metformin and other biguanides on oxidative phosphorylation in mitochondria. *Biochem. J.* **462**, 475–487 (2014). 25017630 10.1042/BJ20140620 PMC4148174

[47] Bridges, H. R. et al. Structural basis of mammalian respiratory complex I inhibition by medicinal biguanides. *Science* **379**, 351–357 (2023). 36701435 10.1126/science.ade3332 PMC7614227

[48] Robb, E. L. et al. Control of mitochondrial superoxide production by reverse electron transport at complex I. *J. Biol. Chem.* **293**, 9869–9879 (2018). 29743240 10.1074/jbc.RA118.003647 PMC6016480

[49] Reczek, C. R. et al. Metformin targets mitochondrial complex I to lower blood glucose levels. *Sci. Adv.* **10**, eads5466 (2024). 39693440 10.1126/sciadv.ads5466 PMC11654692

[50] Iwata, M. et al. The structure of the yeast NADH dehydrogenase (Ndi1) reveals overlapping binding sites for water-and lipid-soluble substrates. *Proc. Natl Acad. Sci. USA* **109**, 15247–15252 (2012). 22949654 10.1073/pnas.1210059109 PMC3458368

[51] Seo, B. B. et al. Molecular remedy of complex I defects: rotenone-insensitive internal NADH-quinone oxidoreductase of *Saccharomyces cerevisiae* mitochondria restores the NADH oxidase activity of complex I-deficient mammalian cells. *Proc. Natl Acad. Sci. USA* **95**, 9167–9171 (1998). 9689052 10.1073/pnas.95.16.9167 PMC21310

[52] Wheaton, W. W. et al. Metformin inhibits mitochondrial complex I of cancer cells to reduce tumorigenesis. *eLife* **3**, e02242 (2014). 24843020 10.7554/eLife.02242 PMC4017650

[53] McElroy, G. S. et al. NAD^+^ regeneration rescues lifespan, but not ataxia, in a mouse model of brain mitochondrial complex I dysfunction. *Cell Metab.* **32**, 301–308 (2020). 32574562 10.1016/j.cmet.2020.06.003 PMC7415718

[54] Molina, J. R. et al. An inhibitor of oxidative phosphorylation exploits cancer vulnerability. *Nat. Med.* **24**, 1036–1046 (2018). 29892070 10.1038/s41591-018-0052-4

[55] Bezawork-Geleta, A., Rohlena, J., Dong, L., Pacak, K. & Neuzil, J. Mitochondrial complex II: at the crossroads. *Trends Biochem. Sci.* **42**, 312–325 (2017). 28185716 10.1016/j.tibs.2017.01.003 PMC7441821

[56] Bisbach, C. M. et al. Succinate can shuttle reducing power from the hypoxic retina to the O2-rich pigment epithelium. *Cell Rep.* **31**, 107606 (2020). 32375026 10.1016/j.celrep.2020.107606 PMC7273505

[57] Liemburg-Apers, D. C., Schirris, T. J., Russel, F. G., Willems, P. H. & Koopman, W. J. Mitoenergetic dysfunction triggers a rapid compensatory increase in steady-state glucose flux. *Biophys. J.* **109**, 1372–1386 (2015). 26445438 10.1016/j.bpj.2015.08.002 PMC4601044

[58] Fletcher, J. W. et al. Recommendations on the use of 18F-FDG PET in oncology. *J. Nucl. Med.* **49**, 480–508 (2008). 18287273 10.2967/jnumed.107.047787

[59] DeFronzo, R., Fleming, G. A., Chen, K. & Bicsak, T. A. Metformin-associated lactic acidosis: current perspectives on causes and risk. *Metabolism* **65**, 20–29 (2016). 26773926 10.1016/j.metabol.2015.10.014

[60] Xiao, S. et al. Lac-Phe mediates the effects of metformin on food intake and body weight. *Nat. Metab.* **6**, 659–669 (2024). 38499766 10.1038/s42255-024-00999-9 PMC11062621

[61] Scott, B. et al. Metformin and feeding increase levels of the appetite-suppressing metabolite Lac-Phe in humans. *Nat. Metab.* **6**, 651–658 (2024). 38499765 10.1038/s42255-024-01018-7 PMC11052712

[62] Coll, A. P. et al. GDF15 mediates the effects of metformin on body weight and energy balance. *Nature* **578**, 444–448 (2020). 31875646 10.1038/s41586-019-1911-y PMC7234839

[63] Kincaid, J. W. et al. The gastrointestinal tract is a major source of the acute metformin-stimulated rise in GDF15. *Sci. Rep.* **14**, 1899 (2024). 38253650 10.1038/s41598-024-51866-2 PMC10803367

[64] Day, E. A. et al. Metformin-induced increases in GDF15 are important for suppressing appetite and promoting weight loss. *Nat. Metab.* **1**, 1202–1208 (2019). 32694673 10.1038/s42255-019-0146-4

[65] Zhang, S.-Y. et al. Metformin triggers a kidney GDF15-dependent area postrema axis to regulate food intake and body weight. *Cell Metab.* **35**, 875–886 (2023). 10.1016/j.cmet.2023.03.014 PMC12272050 37060902

[66] Group, D. P. P. R. Long-term safety, tolerability, and weight loss associated with metformin in the Diabetes Prevention Program Outcomes Study. *Diabetes Care* **35**, 731–737 (2012). 22442396 10.2337/dc11-1299 PMC3308305

[67] Ma, T. et al. Low-dose metformin targets the lysosomal AMPK pathway through PEN2. *Nature* **603**, 159–165 (2022). 35197629 10.1038/s41586-022-04431-8 PMC8891018

[68] Sullivan, L. B. et al. Supporting aspartate biosynthesis is an essential function of respiration in proliferating cells. *Cell* **162**, 552–563 (2015). 26232225 10.1016/j.cell.2015.07.017 PMC4522278

[69] Bailey, C. J. Metformin: historical overview. *Diabetologia* **60**, 1566–1576 (2017). 28776081 10.1007/s00125-017-4318-z

[70] Lannuzel, A. et al. The mitochondrial complex I inhibitor annonacin is toxic to mesencephalic dopaminergic neurons by impairment of energy metabolism. *Neuroscience* **121**, 287–296 (2003). 14521988 10.1016/s0306-4522(03)00441-x

[71] Park, I.-K., Lee, H.-S., Lee, S.-G., Park, J.-D. & Ahn, Y.-J. Antifeeding activity of isoquinoline alkaloids identified in *Coptis japonica* roots against *Hyphantria cunea* (Lepidoptera: Arctiidae) and *Agelastica coerulea* (Coleoptera: Galerucinae). *J. Econ. Entomol.* **93**, 331–335 (2000). 10.1603/0022-0493-93.2.331 10826181

[72] Detzel, A. & Wink, M. Attraction, deterrence or intoxication of bees (*Apis mellifera*) by plant allelochemicals. *Chemoecology* **4**, 8–18 (1993).

[73] Tanner, C. M. et al. Rotenone, paraquat, and Parkinson’s disease. *Environ. Health Perspect.* **119**, 866–872 (2011). 21269927 10.1289/ehp.1002839 PMC3114824

[74] Caparros-Lefebvre, D. & Elbaz, A. Possible relation of atypical parkinsonism in the French West Indies with consumption of tropical plants: a case-control study. *Lancet* **354**, 281–286 (1999). 10440304 10.1016/s0140-6736(98)10166-6

[75] Yin, J., Xing, H. & Ye, J. Efficacy of berberine in patients with type 2 diabetes mellitus. *Metabolism* **57**, 712–717 (2008). 18442638 10.1016/j.metabol.2008.01.013 PMC2410097

[76] Turner, N. et al. Berberine and its more biologically available derivative, dihydroberberine, inhibit mitochondrial respiratory complex I: a mechanism for the action of berberine to activate AMP-activated protein kinase and improve insulin action. *Diabetes* **57**, 1414–1418 (2008). 18285556 10.2337/db07-1552

[77] Maeng, H.-J. et al. P-glycoprotein-mediated transport of berberine across Caco-2 cell monolayers. *J. Pharm. Sci.* **91**, 2614–2621 (2002). 12434406 10.1002/jps.10268

[78] Smolinski, M. P. et al. Discovery of encequidar, first-in-class intestine specific P-glycoprotein inhibitor. *J. Med. Chem.* **64**, 3677–3693 (2021). 33729781 10.1021/acs.jmedchem.0c01826

[79] Zhou, S.-F. Structure, function and regulation of P-glycoprotein and its clinical relevance in drug disposition. *Xenobiotica* **38**, 802–832 (2008). 18668431 10.1080/00498250701867889

[80] Kartaram, S. et al. Plasma citrulline concentration, a marker for intestinal functionality, reflects exercise intensity in healthy young men. *Clin. Nutr.* **38**, 2251–2258 (2019). 30340895 10.1016/j.clnu.2018.09.029

[81] Bailey, S. J. et al. l-Citrulline supplementation improves O_2_ uptake kinetics and high-intensity exercise performance in humans. *J. Appl. Physiol.* **119**, 385–395 (2015). 10.1152/japplphysiol.00192.2014 26023227

[82] Walton, R. G. et al. Metformin blunts muscle hypertrophy in response to progressive resistance exercise training in older adults: a randomized, double-blind, placebo-controlled, multicenter trial: The MASTERS trial. *Aging Cell* **18**, e13039 (2019). 31557380 10.1111/acel.13039 PMC6826125

[83] Konopka, A. R. et al. Metformin inhibits mitochondrial adaptations to aerobic exercise training in older adults. *Aging Cell* **18**, e12880 (2019). 30548390 10.1111/acel.12880 PMC6351883

[84] Barzilai, N., Crandall, J. P., Kritchevsky, S. B. & Espeland, M. A. Metformin as a tool to target aging. *Cell Metab.* **23**, 1060–1065 (2016). 27304507 10.1016/j.cmet.2016.05.011 PMC5943638

[85] He, L. & Wondisford, F. E. Metformin action: concentrations matter. *Cell Metab.* **21**, 159–162 (2015). 25651170 10.1016/j.cmet.2015.01.003

[86] Kajbaf, F., De Broe, M. E. & Lalau, J.-D. Therapeutic concentrations of metformin: a systematic review. *Clin. Pharmacokinet.* **55**, 439–459 (2016). 26330026 10.1007/s40262-015-0323-x

[87] Pentikäinen, P., Neuvonen, P. & Penttilä, A. Pharmacokinetics of metformin after intravenous and oral administration to man. *Eur. J. Clin. Pharmacol.* **16**, 195–202 (1979). 499320 10.1007/BF00562061

[88] Liang, X. & Giacomini, K. M. Transporters involved in metformin pharmacokinetics and treatment response. *J. Pharm. Sci.* **106**, 2245–2250 (2017). 28495567 10.1016/j.xphs.2017.04.078

[89] Shin, H., Schneeweiss, S., Glynn, R. J. & Patorno, E. Trends in first-line glucose-lowering drug use in adults with type 2 diabetes in light of emerging evidence for SGLT-2i and GLP-1RA. *Diabetes Care* **44**, 1774–1782 (2021). 34385345 10.2337/dc20-2926 PMC8385465

[90] Nosadini, R. et al. Effect of metformin on insulin-stimulated glucose turnover and insulin binding to receptors in type II diabetes. *Diabetes Care* **10**, 62–67 (1987). 3552515 10.2337/diacare.10.1.62

[91] Kim, Y.-B. et al. Troglitazone but not metformin restores insulin-stimulated phosphoinositide 3-kinase activity and increases p110β protein levels in skeletal muscle of type 2 diabetic subjects. *Diabetes* **51**, 443–448 (2002). 11812753 10.2337/diabetes.51.2.443

[92] Natali, A. & Ferrannini, E. Effects of metformin and thiazolidinediones on suppression of hepatic glucose production and stimulation of glucose uptake in type 2 diabetes: a systematic review. *Diabetologia* **49**, 434–441 (2006). 16477438 10.1007/s00125-006-0141-7

[93] Hother-Nielsen, O., Schmitz, O., Andersen, P. H., Beck-Nielsen, H. & Pedersen, O. Metformin improves peripheral but not hepatic insulin action in obese patients with type II diabetes. *Eur. J. Endocrinol.* **120**, 257–265 (1989). 10.1530/acta.0.1200257 2648723

[94] Zhang, B., Cao, Y., Qu, Z., Sun, Y. & Tian, X. The impact of metformin on mortality in patients with type 2 diabetes mellitus: a prospective cohort study. *Endocrine* **87**, 136–143 (2025). 39190051 10.1007/s12020-024-04012-x

[95] Forslund, K. et al. Disentangling type 2 diabetes and metformin treatment signatures in the human gut microbiota. *Nature* **528**, 262–266 (2015). 26633628 10.1038/nature15766 PMC4681099

[96] Wang, L. et al. Spatially resolved isotope tracing reveals tissue metabolic activity. *Nat. Methods* **19**, 223–230 (2022). 35132243 10.1038/s41592-021-01378-y PMC10926149

[97] Agrawal, S. et al. El-MAVEN: a fast, robust, and user-friendly mass spectrometry data processing engine for metabolomics. *Methods Mol. Biol.* **1978**, 301–321 (2019). 31119671 10.1007/978-1-4939-9236-2_19

[98] Pang, Z. et al. Using MetaboAnalyst 5.0 for LC–HRMS spectra processing, multi-omics integration and covariate adjustment of global metabolomics data. *Nat. Protoc.* **17**, 1735–1761 (2022). 35715522 10.1038/s41596-022-00710-w

[99] Sebo, Z. & Grant, R. Relevant code for ‘Metformin inhibits mitochondrial complex I in intestinal epithelium to promote glycemic control’. *Zenodo*10.5281/zenodo.15396719 (2026). 10.1038/s42255-026-01530-y PMC13303093 42103929

