# Supplementary material for: Metformin inhibits mitochondrial complex I in intestinal epithelium to promote glycaemic control
Source: Nat Metab. 2026 May 8;8(6):1291–304. doi: 10.1038/s42255-026-01530-y (PMC13303093; doi:10.1038/s42255-026-01530-y)
Supplement: Supplementary file 1 — Supplementary Tables 1 and 2. [file 42255_2026_1530_MOESM1_ESM.pdf]

nature metabolism

Supplementary information https://doi.org/10.1038/s42255-026-01530-y

Metformin inhibits mitochondrial complex I in intestinal epithelium to promote glycaemic control

In the format provided by the authors and unedited

Metabolite	p.value	log2fc	padj
citrulline	5.65E-07	-0.56149	8.13E-05
ribose	1.05E-06	1.39372	8.13E-05
hypoxanthine	0.000133	1.172397	0.006867
glutamic acid	0.000264	0.549237	0.009851
tyrosine	0.00038	-0.23432	0.009851
ornithine	0.000381	-0.20688	0.009851
glucuronic acid	0.000986	-0.368	0.021839
maltotriose	0.001292	0.58123	0.024509
uridine	0.001539	0.379861	0.024509
maltose	0.001581	1.17417	0.024509
hippuric acid	0.001813	0.706574	0.025548
4_hydroxyproline	0.002102	1.737972	0.027154
xylulose NIST	0.003499	0.538115	0.041724
gamma_tocopherol	0.00473	-0.2141	0.052372
conduritol beta expoxide minor	0.008225	-0.25157	0.084996
glutamine	0.010975	-0.25974	0.103248
cytidine_5'_diphosphate	0.011324	0.371976	0.103248
levoglucosan	0.012869	-0.67288	0.110818
arachidonic acid	0.015028	0.364713	0.119321
oxalic acid	0.015396	-0.38206	0.119321
butane_2,3_diol NIST	0.020092	-0.44472	0.148296
3_aminoisobutyric acid	0.021956	-0.22053	0.154687
mannitol	0.025794	-0.23614	0.173827
glycerol_3_galactoside	0.028085	-0.53733	0.181385
cellobiotol	0.030898	0.850903	0.186343
aspartic acid	0.031258	0.50201	0.186343
inosine	0.033078	-0.78496	0.189894
histidine	0.040601	-0.15422	0.224753
threonic acid	0.045473	-0.24774	0.243045
naproxen	0.047358	-0.56577	0.244683

Supplementary Data Table 1. Differentially abundant plasma metabolites before and after metformin treatment in the Rotroff et al. cohort. Metabolites with a raw p-value of less than 0.05 are shown. Statistical significance was determined by two-sided paired t test.

Metabolite	FC	log2(FC)	raw.pval	-log10(p)
citrulline	0.39618	-1.3358	3.85E-06	5.4151
butyrylcarnitine+	0.37729	-1.4063	0.000462	3.3355
glycerol 2/3-phosphate	0.24067	-2.0549	0.000744	3.1286
mevalonic acid	5.0308	2.3308	0.005654	2.2477
IMP	0.33782	-1.5657	0.008913	2.05
5'-deoxyadenosine	0.32686	-1.6132	0.011072	1.9558
D-galactosamine/glucosamine-1/6-phosphate	0.40071	-1.3194	0.013338	1.8749
6-phosphogluconic acid	0.41615	-1.2648	0.015218	1.8176
2-ketobutyric acid	3.2583	1.7041	0.015246	1.8169
deoxycytidine	2.3674	1.2433	0.016841	1.7736
L-NMMA	2.3526	1.2342	0.0193	1.7144
spermidine	0.38805	-1.3657	0.021727	1.663
homoserine / threonine / allothreonine	2.2348	1.1601	0.023776	1.6239
3-phospho-serine	4.3836	2.1321	0.025765	1.589
NADP+	0.32647	-1.615	0.026705	1.5734
purine	0.42249	-1.243	0.026777	1.5722
aconitic acid	0.42708	-1.2274	0.026895	1.5703
UDP	0.42104	-1.248	0.02901	1.5375
valine / norvaline	2.6653	1.4143	0.029853	1.525
L-arginino-succinate	2.3924	1.2585	0.03146	1.5022
glycylglycine	3.743	1.9042	0.032758	1.4847
thiamine diphosphate+	0.35188	-1.5068	0.033357	1.4768
lysine	4.6147	2.2062	0.039226	1.4064
leucine / leucine(iso) / norleucine	2.9931	1.5816	0.039356	1.405
N-acetyl-DL-serine / O-acetyl-L-serine	3.1943	1.6755	0.039875	1.3993
asparagine	2.6279	1.3939	0.04035	1.3942
tyrosine	4.6677	2.2227	0.043162	1.3649
serine	3.3024	1.7235	0.043455	1.362
pantothenic acid	2.1943	1.1337	0.046037	1.3369
methionine	3.9208	1.9711	0.046542	1.3322
arginine	6.7281	2.7502	0.046647	1.3312
2-ketovaleric acid	0.4544	-1.138	0.047297	1.3252
phenylalanine	3.4606	1.791	0.047536	1.323
anserine	0.47704	-1.0678	0.048924	1.3105

Supplementary Data Table 2. Differentially abundant metabolites between VilCre^{metformin} and VilCre:NDI1^{metformin} jejunum. Overnight-fasted mice were oral gavaged with metformin (200 mg kg^{-1}), and jejunum was harvested one hour later. Metabolites with a raw p-value of less than 0.05 are shown. Statistical significance was determined by two-sided unpaired t test.
